# Gut microbiota and gut-kidney axis in kidney diseases: therapeutic potential and perspectives of natural products

**DOI:** 10.3389/fphar.2026.1888944

**Published:** 2026-07-31

**Authors:** Qianwei Wang, Jiahe Zhang, Ruiyan Chen, Junyan Song, Jinye Lv, Zhicheng Zhou, Lijuan Dai

**Affiliations:** 1 Graduate School, Heilongjiang University of Chinese Medicine, Harbin, Heilongjiang, China; 2 Nephrology Department, The First Affiliated Hospital of Heilongjiang University of Chinese Medicine, Harbin, Heilongjiang, China; 3 Graduate School, Wuxi Hospital Affiliated to Nanjing University of Chinese Medicine, Nanjing, Jiangsu, China; 4 Anorectal Department, The First Affiliated Hospital of Anhui University of Chinese Medicine, Hefei, Anhui, China

**Keywords:** gut microbiota, gut-kidney axis, kidney disease, natural products, uremic toxins

## Abstract

Gut microbiota exert core regulatory effects on systemic metabolic homeostasis and immune balance under physiological and pathological conditions. Marked gut dysbiosis occurs during the progression of chronic kidney disease (CKD), characterized by abnormal enrichment of opportunistic pathogens and depletion of beneficial commensal bacteria. This pathological alteration leads to excessive accumulation of uremic toxins including indoxyl sulfate, p-cresyl sulfate and trimethylamine N-oxide (TMAO), as well as sharp reductions in the synthesis of intestinal protective metabolites such as short-chain fatty acids (SCFAs) and indole-3-aldehyde. The imbalance between harmful and protective metabolites persistently triggers renal inflammatory activation and interstitial fibrosis *via* activating multiple signaling pathways, including the renin-angiotensin system, oxidative stress, Toll-like receptor 4, aryl hydrocarbon receptor, IκB/NF-κB, Keap1/Nrf2, NLRP3 inflammasome and ferroptosis. This review systematically summarizes the pathological correlations between gut microbiota disturbance and various renal disorders, namely diabetic kidney disease, membranous nephropathy, lupus nephritis, IgA nephropathy, hypertension-associated nephropathy and dialysis-related renal injury, and clarifies the universal mechanisms and disease-specific characteristics underlying gut-kidney axis-mediated progression of different subtypes of kidney diseases. Natural products represented by natural polysaccharides, traditional Chinese medicine formulas and plant polyphenols are capable of remodeling gut microbiota composition, repairing intestinal mucosal barrier function, balancing microbial metabolic profiles and regulating gut-kidney axis homeostasis, thereby exerting renal and multi-organ protective effects in patients with CKD and dialysis-dependent individuals. Targeted modulation of intestinal microecology and intervention against gut-kidney axis dysfunction using natural products provide novel strategies for early prevention and control of CKD, disease progression retardation and prognosis improvement in dialysis patients, and also lay a theoretical foundation for the development and clinical translational application of novel therapeutic targets for kidney diseases.

## 1 Introduction

Chronic kidney disease (CKD) has emerged as a major global public health burden, with its disease burden escalating rapidly and continuously. Characterized by complex etiologies, insidious onset and poor prognosis, it ranks among the critical diseases threatening human health and increasing healthcare burdens (Amini Khiabani et al., 2023). According to data from the 2021 Global Burden of Disease (GBD) study, the global number of people living with CKD reached nearly 674 million in that year, with a prevalence higher than that of common chronic conditions including diabetes mellitus, chronic respiratory diseases, cardiovascular diseases and osteoarthritis (Ferrari et al., 2024); CKD-related deaths worldwide amounted to 1.5 million in the same year (Foreman et al., 2018). Between 1990 and 2017, the all-age CKD mortality rate globally saw a cumulative increase of 41.5% (Bikbov et al., 2020). Currently, CKD is the third fastest-growing cause of death worldwide and the only non-communicable disease whose age-standardized mortality rate keeps rising persistently, posing an ever-intensifying threat to global public health (Wang et al., 2016). Diabetic kidney disease (DKD), the most prevalent microvascular complication of diabetes mellitus, is the leading cause of end-stage renal disease (ESRD) (Gupta et al., 2023). The incidence of common renal disorders including membranous nephropathy (MN), lupus nephritis (LN), IgA nephropathy (IgAN) and hypertension-complicated CKD has been rising steadily, accompanied by a growing population of dialysis patients with ESRD. Conventional therapeutic strategies mainly focus on localized pathological regulation of renal tissues but fail to fundamentally halt disease progression. Therefore, novel insights into pathogenic mechanisms and breakthrough interventions are urgently required (Ronco et al., 2021; Anders et al., 2020; Xu et al., 2025; Liu XY. et al., 2022).

In recent years, the proposal of the gut-kidney axis theory has updated the traditional understanding of CKD pathogenesis. It confirms close bidirectional regulatory and symbiotic interactions between the intestine and kidney, and identifies gut microbial dysbiosis as a core driver for the initiation and progression of various kidney diseases (Amini Khiabani et al., 2023). Accumulating evidence has demonstrated that patients with DKD, MN, LN, IgAN and other nephropathies all exhibit distinctive gut microbiota disturbances, manifested as depletion of beneficial bacteria, enrichment of pro-inflammatory and uremic toxin-producing microbes, and alterations in microbial community diversity. Such dysbiosis presents disease-specific and stage-specific features, which can serve as potential microbial biomarkers for early diagnosis, disease stratification and prognostic assessment of kidney diseases (Wang Y. et al., 2022; Zhang et al., 2022a; Wang A. et al., 2023; Tang et al., 2023).

Although the pivotal role of gut microbiota in nephropathy has been widely validated, clinically safe, efficient and highly targeted microecological interventions remain scarce. With unique advantages including multi-component properties, multi-target effects, low toxicity and favorable biocompatibility, natural products have emerged as ideal candidates to modulate the gut-kidney axis and prevent as well as treat kidney diseases (Feng et al., 2025). Natural polysaccharides can be selectively utilized by gut microbiota as prebiotics, which directionally reshape microbial composition and facilitate short-chain fatty acid production (Yang et al., 2020). Guided by holistic regulation principles, traditional Chinese medicine formulas correct nephropathy-related gut dysbiosis and block inflammatory and fibrotic signaling cascades (Liu J. et al., 2022). Plant polyphenols restore intestinal barrier integrity, suppress pathogenic bacterial proliferation, alleviate oxidative stress and mitigate renal tissue damage. Collectively, these natural products exert synergistic renal protective effects *via* targeting the gut microbiota-gut-kidney axis, opening up a novel avenue for CKD prevention and management (Foss et al., 2022).

To date, abundant preclinical and clinical data have elucidated the correlations between gut microbiota and distinct nephropathy subtypes, the molecular mechanisms underlying the gut-kidney axis, as well as the microbiota-modulating effects of natural products. Nevertheless, a systematic and integrated theoretical framework has not yet been established. This review comprehensively discusses three core aspects: the specific associations between gut microbial dysbiosis and DKD, MN, LN, IgAN, hypertension-complicated CKD and dialysis-related renal injury, the pivotal pathological pathways whereby the gut-kidney axis mediates nephropathy progression, and the regulatory mechanisms as well as renal protective efficacy of natural polysaccharides, traditional Chinese medicine formulas, plant polyphenols and other natural products against the gut-kidney axis. This study aims to summarize the latest research advances, clarify core scientific questions, and outline future research directions, so as to provide solid theoretical foundations and experimental evidence for gut microecology-based precision prevention and treatment of kidney diseases, as well as the clinical translation of natural products.

## 2 Materials and methods

To establish the literature evidence framework for this review, we systematically searched three core English databases including PubMed, Web of Science and Embase, and supplemented grey literature *via* manual retrieval using Google Scholar. The search strategy combined Medical Subject Headings (MeSH) terms and free-text keywords. The retrieval themes covered chronic kidney disease, gut microbiota/gut microbiome, gut-kidney axis, short-chain fatty acids, and uremic toxins such as indoxyl sulfate, p-cresyl sulfate and trimethylamine N-oxide.

We included multiple CKD subtypes, namely diabetic kidney disease, membranous nephropathy, lupus nephritis, IgA nephropathy, hypertension complicated with chronic kidney disease, as well as renal injuries related to hemodialysis and peritoneal dialysis. Meanwhile, relevant terms concerning natural products, natural polysaccharides, plant polyphenols, traditional Chinese medicine formulas and intestinal targeted interventions were also retrieved.

This retrieval strategy fully collected multi-dimensional research evidence regarding the correlation patterns between gut microbiota structural remodeling and different CKD subtypes, the pathological mechanisms of nephropathy progression mediated by the gut-kidney axis, as well as the renal protective effects exerted by natural products *via* targeted regulation of the intestinal microecology-gut-kidney axis.

## 3 Composition and functions of gut microbiota in healthy state

As the largest microbial colonization niche in the human body, the intestine harbors up to 10^14^ microorganisms, forming a structurally complex and dynamically balanced microecosystem. The physiological functions of gut microbiota extend far beyond traditional nutrient digestion and absorption. By regulating the differentiation of host immune cells, mediating the inter-organ transport of microbial metabolites, and modulating multiple host signaling pathways, gut microbiota profoundly maintains systemic immune homeostasis, metabolic networks and multi-organ functional crosstalk. It serves as a core unit sustaining human physiological balance and general health, and also lays a solid microecological foundation for the bidirectional regulation of the gut-kidney axis (Sanz et al., 2025; Joos et al., 2025; Ross et al., 2024).

Although healthy gut microbiota exhibits obvious individual heterogeneity affected by dietary patterns, lifestyles, geographical environments, age, gender and other factors, ethnic origin has also been confirmed as an independent determinant of gut microbiota profiles—even among populations residing in the same urban environment, individuals of different ethnic backgrounds still present significant differences in microbial alpha diversity, dominant taxa composition and functional metabolic characteristics (Deschasaux et al., 2018). Despite such heterogeneity, the structure of core dominant flora remains relatively conservative with a high level of community diversity. Physiological homeostasis is jointly maintained *via* synergistic metabolism among microbes and bidirectional interactions between microbiota and the host (He et al., 2018; Muralitharan R and Marques, 2021). Currently, academic consensus defines intestinal health as a normal physiological state without obvious organic gastrointestinal lesions or prominent intestinal discomforts (Marco et al., 2026). Bacteria constitute the absolute dominant component (accounting for more than 90%) of healthy intestinal microecology, alongside archaea, fungi, viruses and other microbial components. The intestinal flora presents a hierarchical ecological distribution pattern, in which core microbes are mainly concentrated in a small number of dominant phyla, and clear functional differentiation is formed at the genus and species levels to collectively support host physiological metabolism and immune defense functions (Maiuolo et al., 2024; Van Treuren and Dodd, 2020).

### 3.1 Core flora composition at the phylum level

The intestinal flora of healthy individuals is predominantly composed of five dominant bacterial phyla. Among them, Firmicutes and Bacteroidetes are absolutely predominant, with their combined abundance accounting for approximately 90% of total intestinal bacteria, serving as two core flora maintaining intestinal microecological homeostasis (Van Treuren and Dodd, 2020; Ri et al., 2019). Firmicutes make up 60%–79.4% of the total flora, which are mainly Gram-positive bacteria primarily including Clostridia and Bacilli. This phylum can degrade complex dietary carbohydrates and produce anti-inflammatory active metabolites such as short-chain fatty acids *via* anaerobic fermentation, thus playing a pivotal role in protecting intestinal mucosa and inhibiting excessive inflammatory responses (Maiuolo et al., 2024; Mukhopadhya and Louis, 2025; Tran et al., 2025).

As the second most dominant intestinal flora, Bacteroidetes maintain a stable abundance of 16.9%–25%, represented by anaerobic Gram-negative bacteria mainly including *Bacteroides* and Prevotella. They are centrally involved in intestinal polysaccharide decomposition and carbohydrate metabolic fermentation, and synergistically regulate intestinal microecological balance and host metabolic functions (Joos et al., 2025; Zafar and Saier, 2021; Ilj et al., 2021).

The abundance of Actinobacteria ranges from 2.5% to 3%, most of which are obligate anaerobes. Bifidobacterium, the representative probiotic of this phylum, exerts irreplaceable effects on sustaining intestinal microecological balance and regulating host immune maturation (Xiao et al., 2021; Wang et al., 2024). Under physiological conditions, the abundance of Proteobacteria is merely 1%–10%, and most species belong to opportunistic pathogens. They are suppressed by dominant flora and colonize at a low abundance in healthy hosts, while their abnormal proliferation generally indicates intestinal microecological dysbiosis (Shin et al., 2015; Lin et al., 2023). The abundance of Verrucomicrobia is approximately 0.1%, with Akkermansia muciniphila as its signature dominant species, and fluctuations in its overall abundance are closely correlated with intestinal health status (Ghaffari et al., 2023; Panzetta et al., 2024; Yang and Cong, 2021).

### 3.2 Functional differentiation at genus and species levels

Healthy intestinal flora presents a distinct functional division pattern at the genus and species levels. Beneficial commensal bacteria occupy ecological predominance, whereas pathogenic bacteria and opportunistic pathogens are strictly restricted and maintained at a low-abundance state (Chen et al., 2021). The core beneficial flora mainly include the following species: *Lactobacillus* belonging to Firmicutes can modulate host immune responses, inhibit the proliferation of intestinal harmful bacteria and preserve the integrity of intestinal mucosal barrier (Heeney et al., 2018; Snelson et al., 2019; Zhao et al., 2023). *Faecalibacterium prausnitzii* is capable of secreting potent anti-inflammatory metabolites and enhancing the immune defense capacity of intestinal mucosa (Yang and Cong, 2021). As classic short-chain fatty acid-producing bacteria, Roseburia and Eubacterium rectale participate in the regulation of host glycolipid metabolism and the maintenance of inflammatory homeostasis (Hays et al., 2024).

*Bifidobacterium* of Actinobacteria participates in host nutrient metabolism and immune system development, and can ameliorate glycolipid metabolic disorders and reduce bone loss (Xiao et al., 2021; Ohls et al., 2025; Wang et al., 2026; Cuesta-Marti et al., 2026). *Akkermansia muciniphila* affiliated to Verrucomicrobia can specifically degrade intestinal mucus layer to mediate mucosal remodeling, synthesize active metabolites such as anti-inflammatory lipids, and reinforce intestinal barrier integrity through multiple pathways, thereby stabilizing intestinal microecological homeostasis (Ghaffari et al., 2023; Panzetta et al., 2024; Yang and Cong, 2021). The above core beneficial bacteria collectively lay the functional foundation for intestinal health. Once their abundance is depleted and flora structure becomes imbalanced, the resulting gut-kidney axis disorder will participate in the initiation and progression of kidney diseases.

## 4 Gut microbiota and kidney diseases

### 4.1 Gut microbiota and diabetic kidney disease (DKD)

Diabetic kidney disease (DKD) is the predominant microvascular complication of diabetes mellitus and the leading cause of end-stage renal disease (ESRD) (Thomas et al., 2015). Traditional viewpoints suggest that glucose metabolic disturbance, inflammatory response, oxidative stress imbalance and aberrant activation of the renin-angiotensin-aldosterone system (RAAS) are the core pathogenetic mechanisms of DKD. In recent years, the gut-kidney axis theory has provided a novel research direction for DKD, confirming the bidirectional interaction between gut microbiota and DKD. Accumulating studies have revealed that compared with healthy individuals and patients with uncomplicated diabetes mellitus, DKD patients present characteristic gut dysbiosis featured by depletion of beneficial bacteria, enrichment of pro-inflammatory and toxin-producing microbes, and altered microbial community diversity (Chen et al., 2021; Rukavina Mikusic et al., 2020).

In terms of microbial diversity, gut microbial richness is lower in DKD patients than in healthy controls and patients with uncomplicated diabetes. No consistent difference in alpha diversity is observed between DKD and uncomplicated diabetic patients, whereas beta diversity shows distinct separation (Wang Y. et al., 2022; Shang et al., 2022a; Han et al., 2022a). At the phylum level, DKD patients show decreased abundance of Firmicutes and elevated abundances of Actinobacteria and Proteobacteria (Han et al., 2022a).

Genus-level flora imbalance presents two opposite trends. On the one hand, pro-inflammatory bacteria and opportunistic pathogens are enriched, with increased abundances of *Escherichia-Shigella*, *Akkermansia*, *Clostridium*, *Eisenbergiella* and *Fusobacterium* (Shang et al., 2022a; Lu et al., 2023; Zhang L. et al., 2023), Among them, *Escherichia* and *Eisenbergiella* can serve as signature genera to distinguish DKD from uncomplicated diabetes (Han et al., 2022a; Lu et al., 2023), Continuous enrichment of *Fusobacterium* is closely correlated with DKD progression (Zhang L. et al., 2023). The abundance of *Ruminococcus torques* is negatively correlated with estimated glomerular filtration rate (eGFR) and contributes to renal function impairment (Han et al., 2022a). Moreover, the enrichment of *Escherichia-Shigella* directly damages the intestinal barrier and induces endotoxin translocation (Shang et al., 2022a). On the other hand, a large number of short-chain fatty acid (SCFA)-producing beneficial bacteria are depleted. The abundances of *Prevotella* (especially the subgenus *Prevotella-9*), *Blautia*, *Roseburia*, *Butyricicoccus* and *Anaerostipes* are reduced, which leads to insufficient SCFA synthesis, weakened intestinal barrier protection and impaired anti-inflammatory capacity, thus accelerating DKD progression *via* the gut-kidney axis (Shang et al., 2022a; Han et al., 2022a; Lu et al., 2023; Zhang L. et al., 2023; Zhang et al., 2025).

It has been confirmed that gut microbiota shows stage-specific characteristics during progression from early DKD to advanced DKD (ESRD), and several core genera can be regarded as potential microbial biomarkers for disease staging. In early-stage DKD, pro-inflammatory *Escherichia-Shigella* is enriched and SCFA-producing beneficial bacteria are exhausted, accompanied by a decline in alpha diversity (Wang Y. et al., 2022; Shang et al., 2022a; Zhang L. et al., 2022). Reduced *Agathobacter* abundance can distinguish uncomplicated diabetes from early DKD, as well as early DKD from advanced DKD, acting as a promising early diagnostic biomarker with clinical value (Zhang L. et al., 2023).

In advanced DKD including ESRD, progressive deterioration of renal function triggers characteristic reconstruction of gut microbiota. Compared with early-stage DKD, alpha diversity recovers, and abundances of Proteobacteria, Fusobacteria and Verrucomicrobia increase. Toxin-producing and pro-inflammatory microbes such as Enterobacteriaceae, *Fusobacterium* and *Bilophila* are persistently accumulated. Lipopolysaccharide (LPS) released by these microbes enters the blood circulation, activates the TLR4/NF-κB signaling pathway, promotes secretion of pro-inflammatory cytokines, and further aggravates renal interstitial inflammation and renal fibrosis (Shang et al., 2022a; Linh et al., 2022; Chen et al., 2022).

Patients with stage 5 DKD (i.e., ESRD) show specific enrichment of intestinal flora. Genera including *[Eubacterium]_siraeum_group* and unclassified Ruminococcaceae are specifically enriched and participate in synthesis of uremic toxin precursors such as indole and p-cresol (Shang et al., 2022a). In comparison with ESRD caused by non-diabetic renal diseases, differences are observed in abundances of *Oscillibacter*, *Bilophila* and UBA 1819, which can be used as specific microbial biomarkers for identifying DKD progression to ESRD (Chen et al., 2022).

### 4.2 Gut microbiota dysbiosis and membranous nephropathy (MN)

Membranous nephropathy (MN) is a primary cause of adult nephrotic syndrome, with core pathological features including diffuse subepithelial immune complex deposition on the glomerular basement membrane and basement membrane thickening (Wang YN. et al., 2023; Bey et al., 2024; Miao et al., 2022a). Clinically, idiopathic membranous nephropathy (IMN) accounts for 70%–80% of all cases; it is a renal autoimmune disease targeting podocyte antigens, whereas secondary MN is closely associated with infections, tumors and autoimmune disorders. The gut-kidney axis theory has confirmed that gut microbiota dysbiosis is not merely an accompanying phenomenon of MN, but a pivotal driving factor participating in immune dysregulation and renal damage progression (Ronco et al., 2021; Miao et al., 2022a; Wang YN. et al., 2022).

Patients with IMN present characteristic gut microbial disorders, mainly manifested as imbalanced microbial diversity and bidirectional abundance alterations at phylum and genus levels. Clinical sample studies have shown that gut microbial alpha diversity differs across IMN patients, healthy subjects and CKD patients without nephrotic syndrome, accompanied by specific changes in beta diversity, suggesting impaired structural homeostasis of the overall intestinal microbial community. These microbial alterations are consistent with patterns reported in patients with idiopathic nephrotic syndrome (INS) and MN (Zhang et al., 2020). Reduced abundance and diversity of gut microbiota are also observed in experimental MN rat models, which share consistent dysbiosis trends with IMN patients. Phylum-level analyses identify typical microbial shifts in IMN patients, including increased abundances of Proteobacteria and Fusobacteria and decreased abundance of Firmicutes (Zhang et al., 2022a; Zhang et al., 2020; Shang et al., 2022b). By contrast, an elevated Firmicutes/Bacteroidetes ratio serves as a typical hallmark in MN rat models. The above results collectively verify structural disturbance of gut microbiota under MN conditions from multiple perspectives (Li Z. et al., 2023).

At the genus level, gut microbiota presents a dichotomy characterized by depleted beneficial commensal bacteria and enriched potential pathogens. In terms of beneficial microbes, fecal abundances of *Lactobacillus johnsonii*, *Lactobacillus reuteri*, *Lactobacillus vaginalis*, *Lactobacillus murinus* and *Bifidobacterium animalis* are decreased in both IMN patients and MN model rats (Miao et al., 2024a). Continuous depletion of *Lachnospira*, a core SCFA-producing genus, as well as declines in *Roseburia*, *Megamonas* and *Veillonella* abundances are also widely detected (Zhang et al., 2022a; Zhang et al., 2020; Shang et al., 2022b; Dong et al., 2020). As for potential pathogenic bacteria, enrichment of *Escherichia-Shigella*, *Streptococcus*, *Providencia* and *Myroides* is found in the intestinal flora of IMN patients. Correlation analyses reveal that *Escherichia-Shigella* abundance is negatively correlated with proteinuria levels, while abundances of *Bacteroides* and *Klebsiella* are positively correlated with clinical staging of MN. Synchronous enrichment of *Streptococcus* in intestinal and salivary microbiota further confirms systemic microecological imbalance in MN patients (Shang et al., 2022b; Dong et al., 2020; Shi et al., 2023). Moreover, the gut dysbiosis spectrum of IMN differs from that of diabetic kidney disease, indicating unique disease-specific microecological features (Zhang et al., 2020; Luan et al., 2022).

### 4.3 Gut microbiota dysbiosis and lupus nephritis (LN)

Lupus nephritis (LN) is the most prevalent and severe visceral complication of systemic lupus erythematosus (SLE), affecting approximately 50% of SLE patients. It is pathologically characterized by aberrant autoantibody production and glomerular immune complex deposition, and represents a major risk factor for disability and mortality in SLE patients (Anders et al., 2020; Parikh et al., 2020; Jin et al., 2026). The gut–kidney axis theory has demonstrated distinct alterations in gut microbiota composition in LN patients, who show reduced gut microbial abundance and diversity with lower Chao1 and Shannon indices. Decreased microbial diversity and elevated serum lipopolysaccharide (LPS) levels are shared microecological phenotypes in both SLE and LN patients, while a reduced Firmicutes/Bacteroidetes (F/B) ratio and enrichment of Proteobacteria are microbial features specific to LN (Wang A. et al., 2023; Zeng et al., 2024).

At the phylum level, LN patients show a reduced intestinal F/B ratio, which is closely associated with immune disorders. This reduction is driven primarily by enrichment of *Bacteroides fragilis* within the phylum Bacteroidetes (Wang A. et al., 2023). Meanwhile, Proteobacteria abundance is increased. Bacteria of this phylum secrete LPS to activate TLR4-NF-κB and TLR4-p38MAPK inflammatory signaling pathways, induce interleukin-6 (IL-6) secretion, and contribute to intestinal inflammation, barrier damage and systemic immune activation, making this phylum a hallmark of intestinal microecological imbalance in LN (Shin et al., 2015; Zhao et al., 2021; Salguero et al., 2019).

At the genus and species levels, pathogenic intestinal microbes proliferate excessively in LN patients. Studies confirm that *R. gnavus* (RG) abundance in LN patients is over fivefold higher than in healthy controls. Its abundance correlates positively with SLE/LN disease activity and negatively with serum complement C3 and C4 levels. Moreover, cell wall-derived LPS from *Ruminococcus gnavus* triggers specific IgG antibody production and initiates autoimmune responses, positioning it as a key driver of LN onset, disease activity and clinical relapse (Azzouz et al., 2023; Azzouz et al., 2019). *Streptococcus* abundance correlates positively with SLE activity and acts synergistically with *Veillonella* to amplify systemic inflammatory responses (Li et al., 2019; van den Bogert et al., 2014). Segmented filamentous bacteria (SFB) disrupt microbial community structure, increase renal chemokine levels and promote M2-like macrophage infiltration, ultimately exacerbating renal inflammation and tissue injury (Rahbar Saadat et al., 2019). Regarding beneficial commensal depletion, experimental LN mice show decreased Lactobacillales abundance. Protective strains such as *Lactobacillus reuteri* and species including *Lactobacillus johnsonii* are reduced (Gao et al., 2025; Mu et al., 2017; Pan et al., 2025). Reduced *Lactobacillus* abundance directly downregulates intestinal tight junction proteins such as ZO-1, impairs mucosal barrier integrity, increases intestinal permeability, and enables translocation of intestinal endotoxins and pro-inflammatory cytokines into the bloodstream, further accelerating LN progression (Mu et al., 2017; Valiente et al., 2022).

### 4.4 Gut microbiota and IgA nephropathy (IgAN)

IgA nephropathy (IgAN) is the most prevalent primary glomerulonephritis worldwide. Global prevalence reached 9.3 million cases in 2020 and is projected to rise to 10.2 million by 2030. Incidence is higher in Asian populations than in Caucasian populations, and IgAN is a leading cause of chronic kidney disease in China (Xu et al., 2025; Pattrapornpisut et al., 2021; Schena and Nistor, 2018). IgAN pathogenesis follows a multi-hit mechanism, with aberrant synthesis of galactose-deficient IgA1 (Gd-IgA1) as the core step. This process induces autoantibody production and subsequent immune complex formation that deposits in the glomerular mesangium, ultimately causing renal injury (Stamellou et al., 2023). As a key mediator of mucosal immunity, dysregulated IgA production is closely linked to intestinal mucosal immune dysfunction. Gut microbiota dysbiosis modulates pathogenic IgA synthesis *via* gut-associated lymphoid tissues (GALT) and drives disease progression through the gut–kidney axis (Yang and Palm, 2020; Coppo, 2018).

Compared with healthy individuals, IgAN patients show reduced cellular density and overall diversity of intestinal microbiota (Tang et al., 2022; Zhong et al., 2020). These microbial abnormalities disrupt intestinal microecological homeostasis and increase the risk of pathogenic bacterial colonization (Tang et al., 2023). Several studies have built random forest models based on gut microbiota profiles to reliably distinguish IgAN patients from healthy controls, further confirming gut dysbiosis as a core biological hallmark of the disease (Dong et al., 2022; Noda et al., 2024).

Pathogenic and disease-promoting intestinal microbes are enriched in IgAN patients, including *Escherichia-Shigella*, members of phylum Fusobacteria, *Bacteroides*, *Streptococcus*, *Ruminococcus* and Defluviitaleaceae, with *Escherichia-Shigella*, Fusobacteria and *Bacteroides* as the most representative taxa (Han et al., 2022b; Liang et al., 2022; Shah et al., 2021). *Escherichia-Shigella* abundance correlates positively with urinary red blood cell counts and proteinuria levels, and negatively with estimated glomerular filtration rate (eGFR) (Zhong et al., 2020; Hu X. et al., 2020). Its abundance returns to healthy baseline levels after immunosuppressive therapy-induced clinical remission, supporting its potential as a microbial biomarker for assessing IgAN disease activity (Zhao J. et al., 2022). Additionally, accumulating evidence suggests that mucosal IgA (including Gd-IgA1) production may be linked to the host humoral immune response to intestinal *Escherichia-Shigella*, and this bacterium may contribute to circulating Gd-IgA1 levels (Gao L. et al., 2024).

Meanwhile, mucin-degrading and metabolic enzyme-secreting intestinal bacteria also show abnormal enrichment in IgAN. Increased *A. muciniphila* abundance mediates IgA1 deglycosylation and triggers host autoimmune responses (Gleeson et al., 2024). Enriched intestinal *Flavonifractor plautii* secretes α-galactosidase and α-N-acetylgalactosaminidase, which directly promote formation of Gd-IgA1, a key pathogenic molecule in IgAN (Liang et al., 2022). Furthermore, endotoxin-producing microbes and pathogenic infections contribute to IgAN progression. Increased abundances of Sutterellaceae and Enterobacteriaceae, both typical lipopolysaccharide (LPS)-producing bacteria, have been widely reported (De Angelis et al., 2014). LPS activates Toll-like receptor 4 (TLR4), suppresses mRNA expression of the core β3-galactosyltransferase-specific molecular chaperone (Cosmc) and induces its methylation. As an essential chaperone for IgA1 glycosylation, impaired Cosmc function elevates circulating Gd-IgA1 levels (Akgul et al., 2023; Xiang et al., 2022). With the rising prevalence of type I *H. pylori* (*Helicobacter pylori*) infection, infected patients show elevated proteinuria, blood urea nitrogen and plasma Gd-IgA1 levels. This pathogen exacerbates systemic immune dysregulation by modulating key glycosylation-related molecules, synergistically accelerating IgAN progression (Liu et al., 2020; Zhu et al., 2016).

In contrast to enriched pathogenic microbes, beneficial commensal bacteria are depleted in IgAN patients. Abundances of protective genera including *Bifidobacterium*, *Blautia* and *Prevotella* are decreased. As key probiotics, reduced *Bifidobacterium* levels correlate negatively with proteinuria and hematuria severity (Zhong et al., 2020). Furthermore, reduced levels of IgA protease-secreting commensal bacteria impair degradation of aberrant intestinal IgA immune complexes. Sustained deposition of these complexes consequently aggravates local renal immune-inflammatory damage (Gao L. et al., 2024).

### 4.5 Hypertension complicated with chronic kidney disease (CKD)

A bidirectional vicious cycle links hypertension and chronic kidney disease (CKD). Hypertension induces renal injury, and progressive renal dysfunction in turn causes secondary blood pressure elevation, accelerating progression to end-stage renal disease (ESRD) (Liu XY. et al., 2022; Burnier and Damianaki, 2023; Pugh et al., 2019). Gut–kidney axis studies confirm that intestinal microecological dysbiosis participates actively in core pathological pathways of this condition. Both hypertension and hypertension-related renal damage coincide with reduced gut microbiota abundance and diversity, and fecal microbiota transplantation assays have directly verified that gut microbiota modulates blood pressure phenotypes. Furthermore, the microbial community structure of patients with hypertension-complicated CKD differs from that of patients with isolated hypertension (Li et al., 2017; Karbach et al., 2016; Grylls et al., 2021).

Individuals with hypertension and hypertension-complicated CKD show disrupted intestinal bacterial community homeostasis, characterized by beneficial microbe depletion, harmful bacterial overgrowth and overall microbial community remodeling (Li et al., 2022; Yan et al., 2020). At the phylum level, an elevated Firmicutes/Bacteroidetes ratio is consistently observed in spontaneous hypertension models, angiotensin II-induced hypertension models and hypertensive patients. In high-salt diet-induced salt-sensitive hypertensive rats, gut microbiota restructuring occurs, marked by reduced abundance of beneficial commensal bacteria such as *Lactobacillus*—a key phenotypic feature of hypertension-associated intestinal microecological disturbance (Chakraborty et al., 2018; Okamura et al., 2021; Yang et al., 2015; Adnan et al., 2017). Genus-level microbial alterations show disease-specific patterns. Patients with isolated CKD show enrichment of *Klebsiella* and *Turicibacter*; by contrast, patients with hypertension-complicated CKD have increased *Escherichia* and *Mogibacterium* abundances, alongside reduced *Blautia* and *Clostridium* levels. Abundances of core butyrate-producing beneficial bacteria including *Coprococcus* and *Faecalibacterium* decline progressively with disease worsening, and these changes correlate closely with diastolic blood pressure and serum creatinine levels (Wang P. et al., 2025). The gut microbiota dysbiosis pattern in mouse models of hypertension-complicated CKD aligns closely with clinical observations. 5/6 nephrectomized hypertensive rats show increased intestinal *Bacteroides* and decreased *Lactobacillus* abundance, and *Lactobacillus* abundance changes correlate with urinary protein excretion (Yoshifuji et al., 2016). In adenine-induced hypertensive CKD rats, intestinal *Ligilactobacillus* and *Duncaniella* are decreased, while *Eubacterium* and *Schaedierella* are increased (Tain et al., 2023). In addition, hypertensive populations show enrichment of lactic acid-producing bacteria such as *Prevotella*, *Klebsiella*, *Turicibacter* and *Streptococcus*, alongside reduced short-chain fatty acid-synthesizing beneficial microbes including Clostridiaceae, *Bacteroides* and *Akkermansia* (Li et al., 2017; Lupu et al., 2023; Wu et al., 2021). Among these, *Oxalobacter formigenes* and *Veillonella parvula* serve as specific differential microbial biomarkers for hypertensive nephropathy (Li et al., 2022).

Intestinal fungal microecological imbalance is evident in patients with hypertension-complicated CKD, alongside altered community structure and species abundance. In these patients, intestinal abundances of *Apiotrichum*, *Cystobasidium* and *Saccharomyces* are increased, while *Candida* shows a decreasing trend, alongside elevated overall fungal community diversity. Correlation analyses show that *Apiotrichum* and *Saccharomyces* abundances are associated with renal function indicators, and *Septoria* levels correlate closely with diastolic blood pressure, indicating that intestinal fungal microecological disturbance also contributes to the pathological progression of hypertension-complicated CKD (Qiu et al., 2023).

### 4.6 Changes in gut microbiota among dialysis patients with end-stage renal disease

#### 4.6.1 Gut microbiota characteristics of hemodialysis patients

Distinct structural remodeling of intestinal microecology occurs in end-stage renal disease (ESRD) patients receiving hemodialysis (Tourountzis et al., 2022). Multiple factors including internal environmental disturbance under uremic conditions, dialysis therapy itself, strict dietary restrictions and long-term pharmaceutical interventions jointly disrupt gut microbial homeostasis. In turn, dysregulated gut microbiota further mediates uremic toxin accumulation and systemic microinflammation, and plays a critical role in the development of complications and disease progression in hemodialysis patients (Simões-Silva et al., 2018; Zhang X. et al., 2023; Frąk et al., 2024; Rusu et al., 2026).

Compared with healthy individuals, no consistent difference is observed in gut microbial alpha diversity among hemodialysis patients, whereas beta diversity shows clear separation, resulting in structural heterogeneity across microbial communities. Dialysis intervention can reshape the intestinal microecological landscape of ESRD patients, and these microbial structural alterations show dialysis modality-dependent features (Wu et al., 2023; Luo et al., 2021). At the phylum level, the intestinal flora of hemodialysis patients presents characteristic shifts relative to healthy controls, mainly manifested as elevated abundances of Firmicutes and Proteobacteria, decreased abundance of Bacteroidetes, and enrichment of Actinobacteria in some patients (Wu et al., 2023; Hobby et al., 2019; Chao et al., 2023; Gao Q. et al., 2024). Notably, gut microbial profiles differ between pediatric and adult hemodialysis patients; pediatric patients harbor higher Bacteroidetes abundance than healthy subjects and lower Proteobacteria abundance than peritoneal dialysis (PD) patients (Wu et al., 2023; Crespo-Salgado et al., 2016).

Gut dysbiosis is more evident at the genus level. The abundances of *Blautia obeum*, *Escherichia* and *Enterococcus* tend to increase, while *Bifidobacterium* is depleted (Zhang X. et al., 2023; Wu et al., 2023; Chao et al., 2023; Gao Q. et al., 2024; Stadlbauer et al., 2017). Elevated abundances of the *Escherichia-Shigella* complex and *Klebsiella* are closely associated with the incidence of adverse clinical events (Shi et al., 2022; Lin TY. et al., 2021). Meanwhile, core butyrate-producing microbes such as *Faecalibacterium*, as well as SCFA-synthesis-related genera including *Succinivibrio* and *Anaerospirillum*, are reduced. These microbial structural abnormalities are strongly correlated with long-term adverse clinical outcomes in hemodialysis patients (Luo et al., 2021).

#### 4.6.2 Gut microbiota profiles of peritoneal dialysis (PD) patients

Peritoneal dialysis serves as an essential renal replacement therapy for patients with end-stage renal disease (ESRD) (Li et al., 2025a). During long-term peritoneal dialysis treatment, multiple factors including sustained glucose load and clinical iron supplementation disrupt the native homeostasis of intestinal microecology and induce characteristic gut microbiota dysbiosis (Wang Q. et al., 2025; Stepanova, 2023). Such dysbiosis is mainly manifested as reduced microbial community diversity, decreased abundance of beneficial flora involved in short-chain fatty acid synthesis, and excessive proliferation of harmful bacteria, accompanied by host metabolic disorders and uremic toxin accumulation. Intestinal microecological imbalance not only aggravates systemic microinflammation and extra-renal organ damage, but also contributes to peritoneal fibrosis progression *via* gut-peritoneum crosstalk, and increases the risk of peritonitis and cardiovascular complications. It is a critical factor limiting the therapeutic efficacy of peritoneal dialysis and long-term clinical prognosis of patients (March et al., 2020; Bello et al., 2022). Differences in gut microbial structure exist between PD patients and healthy individuals. PD patients show decreased alpha diversity, alongside reductions in both microbial species richness and evenness (Crespo-Salgado et al., 2016; Li J. et al., 2023; Bao et al., 2022). Clear community separation in beta diversity is observed relative to healthy subjects, with heterogeneity in overall microbial structure (Luo et al., 2021; Bao et al., 2022; Gao et al., 2020; Hu J. et al., 2020). Compared with non-dialyzed ESRD patients and hemodialysis patients, PD patients present more severe gut microbiota dysbiosis. Additionally, divergences are found in dominant flora composition, pathogenic bacterial colonization traits and microbial functional metabolic phenotypes between PD and hemodialysis patients, indicating that dialysis modality is a core determinant shaping the intestinal microecological landscape of ESRD patients (Luo et al., 2021; Hu J. et al., 2020).

At the phylum, class and order taxonomic levels, intestinal abundances of Firmicutes and Actinobacteria are downregulated, while Proteobacteria show elevation; nevertheless, Bacteroidetes abundance variation shows some heterogeneity across studies. These declining trends of dominant flora are more evident in pediatric PD patients, and Proteobacteria proliferation is closely correlated with oral iron supplementation. At the class level, PD patients are characterized by decreased Clostridia and enriched Gammaproteobacteria. At the order level, reduced Clostridiales abundance and expanded Enterobacteriales distribution are commonly detected (Simões-Silva et al., 2018; Crespo-Salgado et al., 2016; Stadlbauer et al., 2017; Li J. et al., 2023; Bao et al., 2022; Hu J. et al., 2020).

Consistent abnormal remodeling of intestinal microbiota is also identified at the family, genus and species levels. At the family level, abundances of intestinal protective flora including Bifidobacteriaceae, Lactobacillaceae and Lachnospiraceae are generally decreased, while Enterobacteriaceae are expanded—a core hallmark of gut dysbiosis in PD patients (Simões-Silva et al., 2018; Crespo-Salgado et al., 2016). At the genus level, SCFA-producing beneficial bacteria such as *Faecalibacterium*, *Roseburia* and *Bifidobacterium* are universally depleted, whereas toxin-producing bacteria represented by *Escherichia-Shigella* proliferate widely. Notably, the genus *Blautia* is a unique dominant taxon in PD populations (Li J. et al., 2023; Bao et al., 2022; Peng et al., 2023; Wang J. et al., 2023). At the species level, persistently low *F. prausnitzii* abundance and high *Escherichia coli* colonization density are the most stable characteristic biomarkers during intestinal microecological remodeling in peritoneal dialysis patients (Li J. et al., 2023).

### 4.7 Horizontal comparison of gut microbiota dysbiosis among different subtypes of chronic kidney disease

**FIGURE 1:**
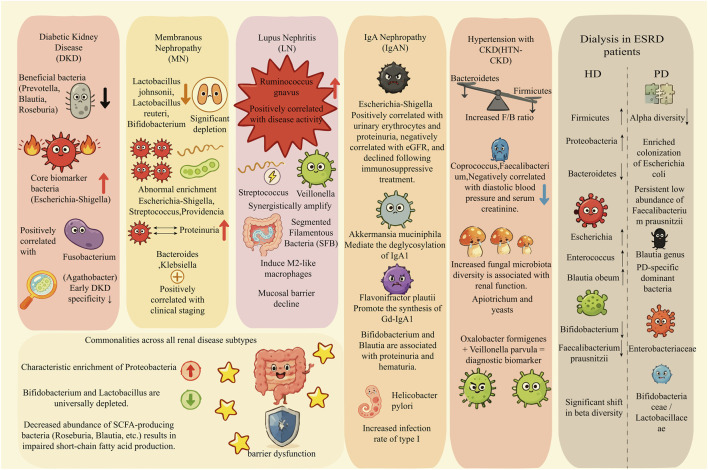
Six independent illustration panels are arranged in order to display the characteristic gut dysbiosis phenotypes of diabetic kidney disease, membranous nephropathy, lupus nephritis, IgA nephropathy, hypertension complicated with CKD, and hemodialysis/peritoneal dialysis-associated renal injury, so as to intuitively reflect the unique patterns of beneficial bacteria depletion and pathogenic bacteria enrichment corresponding to each renal disease subtype. The common features of gut microbiota imbalance among all chronic kidney disease subtypes are summarized at the bottom, which distinctly presents the specificity and universal rules of gut microbial fingerprints in various kidney diseases.

By integrating research outcomes on gut microbiota alterations in diabetic kidney disease (DKD), membranous nephropathy (MN), lupus nephritis (LN), IgA nephropathy (IgAN), hypertension complicated with chronic kidney disease (CKD) and dialysis patients with end-stage renal disease, consistent common features of gut microbiota dysbiosis have been confirmed across various CKD subtypes. All these diseases are universally characterized by extensive depletion of beneficial commensal bacteria such as *Bifidobacterium* and *Lactobacillus*, specific enrichment of Proteobacteria, and insufficient short-chain fatty acid (SCFA) synthesis driven by reduced core SCFA-producing genera including *Blautia* and *Roseburia*. Such pathological changes further impair intestinal barrier function, induce endotoxin translocation, and accelerate renal inflammation and fibrosis progression through the gut-kidney axis. Meanwhile, each CKD subtype harbors distinct disease-specific microbial biomarkers. Specifically, *Escherichia-Shigella* is identified as the core signature genus of DKD (Shang et al., 2022a); *R. gnavus functions as the key pathogenic driver of LN development* (Azzouz et al., 2023; Azzouz et al., 2019); *Escherichia-Shigella also acts as the core microbial biomarker for assessing IgAN disease activity* (Zhong et al., 2020; Hu X. et al., 2020); MN features enrichment of *Escherichia-Shigella* and *Streptococcus* (Shang et al., 2022b; Dong et al., 2020; Shi et al., 2023); moreover, patients with hypertension-complicated CKD and ESRD dialysis patients present microbial phenotypes specific to their corresponding disease states and treatment regimens. These shared pathogenic patterns and disease-specific biomarkers provide an important theoretical basis for further clarifying gut-kidney axis-mediated renal disease progression, enabling precise subtyping and clinical diagnosis of kidney diseases, and delivering targeted microecological intervention strategies (Figure 1).

## 5 Remodeling of gut microbiota in kidney diseases by natural products

### 5.1 Definition of natural products

Natural products are a general term for bioactive chemical substances synthesized by organisms in nature. As a major category, natural polysaccharides are high-molecular polymers formed by linking more than ten monosaccharides *via* glycosidic bonds, ranking among key biological macromolecules alongside nucleic acids, proteins and lipids. They are characterized by good biocompatibility, biodegradability and low immunogenicity. Not degraded by human endogenous enzymes, natural polysaccharides can reach the distal intestine and be selectively utilized by intestinal flora. They have nutritional value and multiple pharmacological activities including prebiotic, anti-inflammatory and antioxidant effects, and represent a core research focus in the natural product field (Feng et al., 2024; Guo et al., 2024; Fang et al., 2019).

### 5.2 Core signaling pathways for natural products to regulate the gut microbiota-gut-kidney axis

A vicious cycle exists between intestinal microecological imbalance and renal function impairment in chronic kidney disease (CKD). Structural disorders and metabolic dysfunction of gut microbiota disrupt intestinal barrier integrity, leading to massive entry of gut-derived toxins including lipopolysaccharide (LPS), indoxyl sulfate (IS), p-cresyl sulfate (p-CS) and trimethylamine N-oxide (TMAO) into the bloodstream. These toxins accelerate renal injury progression by activating local renal inflammatory, oxidative stress and fibrotic signaling cascades (Rysz et al., 2021). Natural products exert protective effects on the gut-kidney axis by intervening in multiple key nodes within this pathological chain. Based on available research evidence, their regulatory mechanisms can be categorized into four core signaling hierarchies.

In the hierarchical inhibition of inflammatory signaling pathways, the TLR4/NF-κB pathway serves as the central hub whereby natural products modulate gut-kidney axis-mediated inflammatory responses. Pharmacological studies have demonstrated that TAK-242, a selective TLR4 inhibitor, attenuates sepsis-induced acute kidney injury by blocking the activation of this pathway, inhibiting the nuclear translocation of downstream NF-κB and suppressing NLRP3 inflammasome activation (Xia et al., 2024; Guo et al., 2026). Statins also exert anti-inflammatory effects *via* inhibiting the TLR4/MyD88/NF-κB axis, which further validates this pathway as a key therapeutic target in the regulation of renal inflammation (Kong et al., 2016). Under CKD conditions, gut-derived LPS activates NF-κB *via* the TLR4/MyD88 axis, induces pro-inflammatory factors such as TNF-α and IL-6, and further exacerbates renal damage (Di Vincenzo et al., 2024). Natural products inhibit this pathway mainly through two distinct patterns. The first is microbiota-dependent indirect inhibition, which reshapes gut microbiota composition and reduces gut-derived LPS production to block TLR4/NF-κB activation. Recent studies have demonstrated that *Cordyceps cicadae* polysaccharides (CCP) and *Bupleurum chinense* polysaccharides (BCP) selectively enrich SCFA-producing and butyrate-producing bacteria respectively, thereby reducing LPS translocation and suppressing this signaling pathway (Yang et al., 2020; Feng et al., 2019). Curcumin also lowers LPS generation by inhibiting opportunistic pathogens in hyperuricemic nephropathy models (Xu X. et al., 2021). The second is direct targeted inhibition, in which certain natural products act directly on key molecules of the TLR4 signaling cascade. Resveratrol suppresses renal TLR4/NF-κB pathway activation and decreases serum LPS and inflammatory factor levels in doxorubicin-induced CKD rats (Xu X. et al., 2021). The traditional Chinese medicine compound QRXZF inactivates this pathway directly by reducing circulating LPS concentrations (Gao et al., 2021).

The aryl hydrocarbon receptor (AhR) pathway represents another pivotal inflammatory signaling cascade. Indole derivatives from gut microbiota-mediated tryptophan metabolism activate AhR, and excessive AhR activation promotes renal inflammation and fibrosis (Stockinger et al., 2021). As a representative tryptophan-derived microbial metabolite, indolepropionic acid (IPA) is synthesized *via* the reductive aromatic amino acid pathway mediated by phenyllactate dehydratase in specific commensals such as *Clostridium* sporogenes; genetic manipulation studies have verified its core role in maintaining intestinal mucosal barrier integrity and modulating systemic immune homeostasis (Dodd et al., 2017). Justicinoside A (BSA) directly restrains AhR signaling activation and alleviates renal inflammation by modulating the NF-κB and Nrf2 pathways. In addition, *Lactobacillus johnsonii* mitigates CKD-related renal injury by elevating serum indole-3-aldehyde (IAId) to inhibit excessive AhR signaling (Miao et al., 2024b; Miao et al., 2022b; Miao et al., 2020; Li et al., 2024).

In terms of bidirectional regulation of gut microbial metabolites, restoration of the SCFA axis is a vital mechanism underlying the renoprotective effects of natural products. SCFA-producing bacteria are universally depleted in CKD. Natural products can enrich SCFA-synthesizing flora and elevate systemic SCFA levels, thereby exerting anti-inflammatory and renoprotective effects through the SCFA-GPR receptor signaling pathway. Mechanistically, SCFAs mainly function by activating G-protein coupled receptors including GPR43 (FFAR2) and GPR41 (FFAR3), which further mediate the regulation of intestinal barrier integrity, inflammatory cascades and systemic metabolic homeostasis (Dodd et al., 2017). Resveratrol enriches beneficial genera including *Lactobacillus* and *Faecalibaculum* and increases intestinal SCFA content in db/db diabetic nephropathy mice. Fecal microbiota transplantation assays have confirmed that the SCFA-gut microbiota axis mediates its anti-fibrotic efficacy (Yan et al., 2024; Cai et al., 2020). Punicalagin raises cecal propionate and butyrate levels in high-fat diet-induced diabetic mice, and the abundance of enriched SCFA-producing bacteria is negatively correlated with renal function indicators (Hua et al., 2022). Fushen Granules (FSG) indirectly facilitate SCFA synthesis by upregulating *Bacteroides* abundance (Lin W. et al., 2021).

Inhibition of the TMAO/TMA pathway is also an essential therapeutic target. As a typical uremic toxin produced by intestinal microbial metabolism of dietary choline, TMAO triggers ferroptosis and profibrotic factor secretion in renal tubular epithelial cells. It can also aggravate renal tissue damage by inducing oxidative stress, activating the NLRP3 inflammasome and promoting the secretion of pro-inflammatory cytokines through multiple downstream signaling cascades (Dodd et al., 2017). Resveratrol blocks the conversion of TMA to TMAO and reduces the plasma TMAO/TMA ratio in offspring hypertensive rats with maternal-derived CKD (Hsu et al., 2020). Suyin Jiedu Granules downregulate TMAO-synthesis-related microbiota, lower circulating TMAO levels and suppress ferroptosis (Ge et al., 2024).

For the regulation of indole-derived and phenolic toxins, indoxyl sulfate (IS) and p-cresyl sulfate (p-CS) are mainly synthesized by intestinal flora *via* tryptophan and tyrosine catabolism. Isoquercitrin (ISO) reduces indole biosynthesis (the IS precursor) by targeting intestinal bacterial complex I activity (Wang et al., 2020). Epigallocatechin gallate (EGCG) decreases Firmicutes and Clostridiales abundance to restrain tyrosine metabolism and alleviate p-CS accumulation (Unno and Ichitani, 2022). Shenshuaining Granules (SSN) reduce serum IS levels in peritoneal dialysis patients (Chen et al., 2018).

With regard to suppression of oxidative stress and cell death pathways, activation of the Nrf2/ARE antioxidant pathway is the core approach by which natural products counteract oxidative damage in CKD. Luteolin activates the SIRT1/6-Nrf2/ARE signaling axis to enhance antioxidant enzymes such as SOD and CAT and reduce MDA levels (Wang et al., 2011; Khayyat et al., 2025; Yu et al., 2024a). Resveratrol elevates the plasma L-arginine/ADMA ratio, decreases renal 8-OHdG levels and improves nitric oxide bioavailability. Fucoidan oligosaccharides activate the renal Nrf2 pathway to alleviate cisplatin-induced oxidative injury (Chen et al., 2016; Zhang et al., 2022c).

Inhibition of the ferroptosis pathway has become a novel research focus in CKD pathogenesis. Luteolin binds directly to nuclear receptor Nr4a1 and blocks ferroptosis-mediated lipid peroxidation and mitochondrial damage by regulating the Nr4a1-Slc7a11-GPX4 signaling cascade (Ye et al., 2025). Suyin Jiedu Granules inhibit ferroptosis in renal tubular epithelial cells by lowering TMAO concentrations (Ge et al., 2024).

Suppression of the NLRP3 inflammasome pathway is also critical for the anti-inflammatory and renoprotective actions of natural products. Luteolin from Hongshenguo Fruit Extract (HGGG) restrains excessive renal NLRP3 inflammasome activation by restoring gut microbial homeostasis; furthermore, SCFAs show interactive crosstalk with the NLRP3 pathway (Yuan et al., 2021).

In terms of intestinal barrier maintenance and systemic metabolic regulation, intact intestinal barrier function serves as the physical foundation for gut-kidney axis protection. Natural products fundamentally block the transmission of gut-derived pathogenic signals to the kidney by upregulating tight junction protein expression and repairing damaged intestinal mucosal structure. Luteolin repairs impaired intestinal barriers by increasing intestinal epithelial Claudin-1 and ZO-1 expression (Yuan et al., 2021). Resveratrol restores ZO-1 and Claudin-1 expression and reduces intestinal leakage in db/db mice (Cai et al., 2020). Curcumin improves intestinal barrier function and upregulates tight junction proteins in hyperuricemic nephropathy rat models (Xu X. et al., 2021). The combined application of *Astragalus membranaceus* and *Salvia miltiorrhiza* repairs intestinal barriers *via* enriching butyrate-producing bacteria (Han et al., 2021). In addition, certain natural products exert indirect renoprotective effects by modulating host systemic metabolism. While inhibiting renal tubulointerstitial fibrosis by specifically suppressing profibrotic signaling pathways including TGF-β/Smad3 and Wnt/β-catenin, Alisol B 23-acetate (ABA) also reverses the abnormal abundance of twelve hypertension-related intestinal bacterial genera in 5/6 nephrectomized rats (Chen et al., 2020).

Notably, the aforementioned signaling pathways do not operate independently, but form a highly interconnected regulatory network that collectively mediates the multi-target renoprotective effects of natural products along the gut–kidney axis. At the upstream level, gut microbiota dysbiosis and intestinal barrier disruption serve as the initiating links of the pathological process. Elevated gut-derived LPS, uremic toxins and microbial metabolites can simultaneously trigger multiple downstream signaling cascades in renal tissues after entering the systemic circulation. The TLR4/NF-κB axis, as the core inflammatory hub, has extensive crosstalk with other signaling modules: it promotes NLRP3 inflammasome assembly to amplify inflammatory cascades, and interacts with the AhR pathway to synergistically exacerbate renal inflammation and fibrosis. In contrast, the SCFA-GPR signaling axis exerts negative regulatory effects on both the TLR4/NF-κB pathway and the NLRP3 inflammasome, constituting an endogenous anti-inflammatory feedback loop. At the level of oxidative stress and cell death regulation, the Nrf2/ARE antioxidant pathway antagonizes NF-κB-mediated pro-inflammatory activation, and AhR signaling also coordinates the balance between inflammation and oxidative stress by modulating Nrf2 activity. The TMAO–ferroptosis axis further forms a feedforward pathological cycle with inflammatory pathways: ferroptosis of renal tubular epithelial cells activates local inflammatory cascades including the NLRP3 inflammasome, which in turn exacerbates intestinal barrier injury and gut microbiota dysbiosis, ultimately amplifying overall damage along the gut–kidney axis.

**FIGURE 2:**
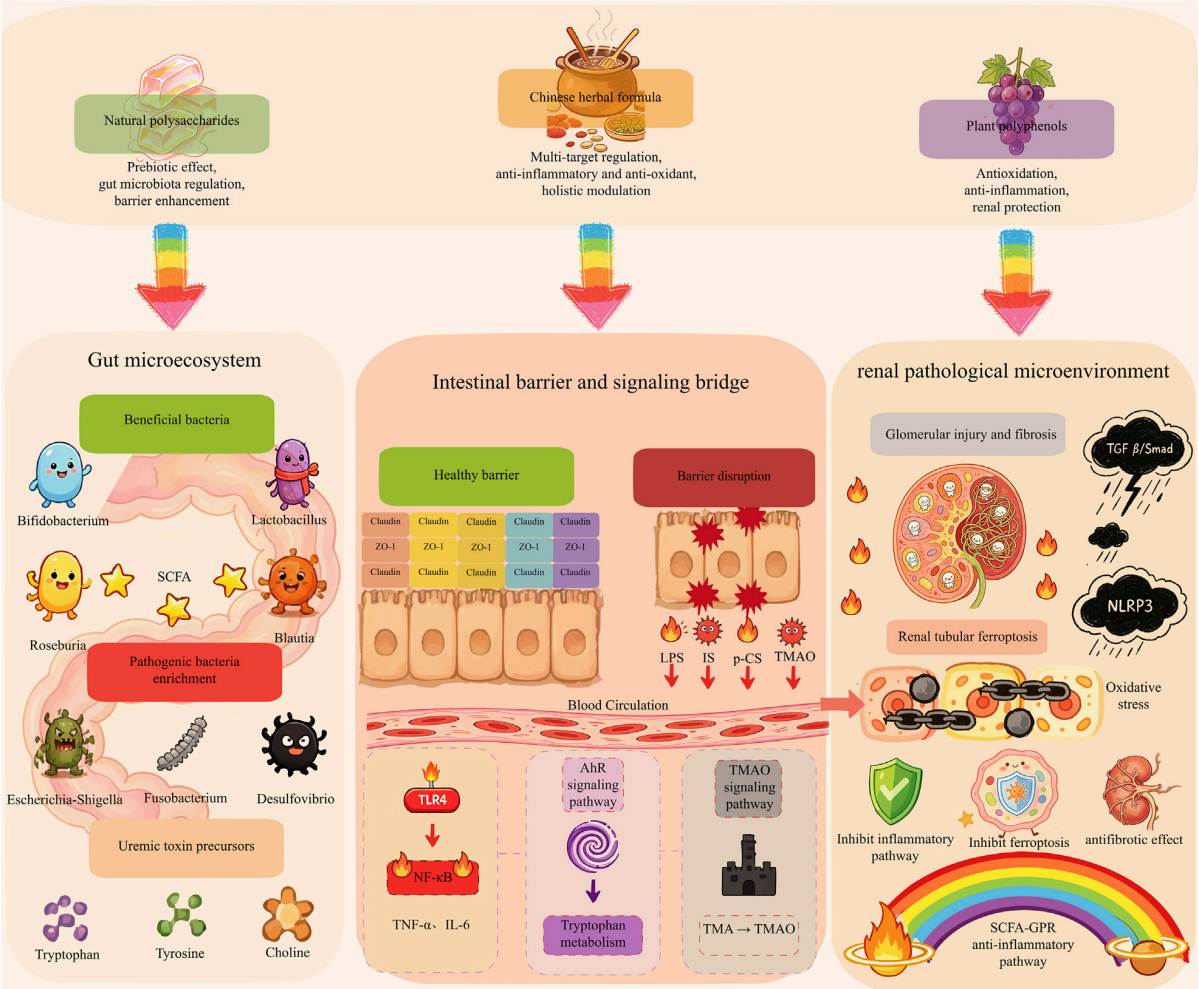
This diagram consists of three core components: intestinal microecology, intestinal barrier together with gut-kidney axis-related signaling pathways, and pathological microenvironment in the kidney. Chronic kidney disease triggers gut microbiota dysbiosis characterized by decreased beneficial bacteria and expanded pathogenic bacteria, which disrupts tight junction proteins including Claudin and ZO-1 and further induces intestinal barrier injury. Gut-derived toxins such as lipopolysaccharide, indoxyl sulfate, p-cresyl sulfate and trimethylamine N-oxide enter the blood circulation, subsequently activating TLR4/NF-κB, AhR, NLRP3, ferroptosis and other signaling pathways, and ultimately triggering renal inflammation, oxidative stress and renal interstitial fibrosis. Natural polysaccharides, traditional Chinese medicine formulas and plant polyphenols are capable of remodeling gut microbiota composition, repairing damaged intestinal barrier and blocking pathological signal transmission, thereby exerting renoprotective effects *via* restoring the homeostasis of the gut-kidney axis.

In conclusion, the regulatory effects of natural products on the gut microbiota-gut-kidney axis are not simple linear actions *via* a single pathway, but synergistic renoprotective effects achieved through multi-layered mechanisms: gut microbiota structural remodeling, microbial metabolite rebalancing, hierarchical control of inflammatory signals, intestinal barrier function restoration and cell death blockade. Among these, the TLR4/NF-κB signaling pathway acts as a core bridge connecting gut-derived stimuli and renal inflammatory responses, and occupies a pivotal position in the regulatory network of multiple natural products. Moreover, the discovery of the TMAO-ferroptosis axis, AhR-tryptophan metabolism axis and SCFA-GPR inflammatory regulatory axis has further enriched the multi-dimensional understanding of gut-kidney axis protective mechanisms mediated by natural products (Figure 2).

### 5.3 Prebiotic-like renoprotective effects of polysaccharides

As typical prebiotic-type natural bioactive components, natural polysaccharides synergistically suppress renal inflammatory responses and renal tubulointerstitial fibrosis progression by targeted remodeling of gut microbial homeostasis, repairing the intestinal mucosal barrier and regulating gut-kidney axis signal transduction, thereby improving renal function and alleviating renal tissue damage. Their renoprotective efficacies have been validated in multiple renal disease models including diabetic kidney disease and chemotherapy-associated renal injury.

*Cordyceps cicadae* polysaccharides rectify gut microbiota dysbiosis in diabetic nephropathy rats, and elevate abundances of beneficial genera such as *Lactobacillus*, *Bacteroides* and *Akkermansia*. It exerts dual protective effects against renal inflammation and renal tubulointerstitial fibrosis by inhibiting aberrant activation of the TLR4/NF-κB inflammatory pathway and blocking the profibrotic TGF-β1/Smad signaling axis (Yang et al., 2020). Sanzi Guben polysaccharides ameliorate gut microbial disturbance in diabetic nephropathy mice, and reduce abundances of harmful Gram-negative bacteria including Proteobacteria, *Klebsiella* and *Escherichia-Shigella* to lower plasma LPS levels, thus restraining activation of the TLR4/NF-κB/NLRP3 inflammatory cascade and relieving inflammatory renal injury (Wang F. et al., 2023). *Bupleurum chinense* polysaccharides enhance gut microbial diversity and reverse the Firmicutes/Bacteroidetes ratio imbalance in streptozotocin-induced diabetic nephropathy mice. It specifically enriches butyrate-producing beneficial bacteria such as *Roseburia* and *Eubacterium* to repair the intestinal mucosal barrier and reduce LPS translocation, and simultaneously alleviates intestinal and renal inflammation by suppressing the LPS/TLR4/NF-κB pathway (Feng et al., 2019). Moutan bark polysaccharides concurrently ameliorate hyperglycemia and renal function impairment in diabetic nephropathy rats, decrease serum creatinine, blood urea nitrogen and urinary albumin-to-creatinine ratio, and mitigate renal pathological lesions. Meanwhile, it remodels disordered gut microbiota structure, upregulates abundances of beneficial bacteria including *Akkermansia*, *Lactobacillus* and butyrate-producing Muribaculaceae, elevates intestinal short-chain fatty acid levels and repairs the damaged intestinal mucosal barrier (Zhang M. et al., 2022). Beyond interventions targeting diabetic kidney disease, fucoidan oligosaccharides exert specific protective effects against chemotherapy-associated renal injury. They increase intestinal abundances of *Lactobacillus johnsonii* and *Lactobacillus reuteri* in cisplatin-treated mice, promote synthesis of fatty acid hydroxy fatty acid esters, and further activate the renal Nrf2 antioxidant pathway *via* these metabolites. This process boosts activities of antioxidant enzymes including superoxide dismutase and catalase, reduces malondialdehyde content, and alleviates cisplatin-induced renal oxidative damage, with potential synergistic multi-organ protective properties (Zhang et al., 2022c) (Table 1).

**TABLE 1:** Effects of polysaccharides on gut microbiota in chronic kidney disease (CKD).

Patient/animal model	Intervention protocol	References	Dosage	Study type	Changes in relative abundance of gut microbiota		Main effects
					Increased	Decreased	
Diabetic nephropathy rat model	Intervention with *Cordyceps cicadae* polysaccharides (CCP); metformin (DMBG) served as positive control; low, medium and high CCP dose groups were set for continuous intervention for 4 weeks	Yang et al. (2020)	CCP: 75 mg/kg, 150 mg/kg, 300 mg/kg	Animal experiment	Verrucomicrobia, *Actinobacteria, Lactobacillus, Bacteroides, Akkermansia, Ruminococcus, Oscillospira,* Prevotellaceae	Proteobacteria, Deferribacteres	Remodel gut microbiota structure, upregulate the abundance of probiotics, and inhibit the proliferation of LPS-producing pathogenic bacteria
Diabetic nephropathy mice	Intragastric administration of Sanzi Guben polysaccharides (SZP); metformin (MET) served as positive control; continuous administration for 8 weeks	Wang et al. (2023d)	SZP 500 mg/kg; MET 300 mg/kg	Animal experiment	Verrucomicrobia, *Akkermansia*	Proteobacteria, *Klebsiella, Escherichia-Shigella, Marvinbryantia*	Ameliorate gut microbiota dysbiosis, reduce levels of Gram-negative bacteria and LPS, and relieve insulin resistance and renal function damage
Diabetic nephropathy mice	Oral intervention with two types of Bupleurum chinense polysaccharides (BCP, BPs); metformin (DMBG) served as positive control; continuous administration for 6 weeks	Feng et al. (2019)	BCP and BPs: both 60 mg/kg; STZ modeling dose: 100 mg/kg	Animal experiment	BCP enriched Ruminococcaceae, Rikenellaceae and *Roseburia*; BPs upregulated Tenericutes and *Turicibacter*	—	Improve gut microbiota disturbance, repair intestinal barrier; reduce blood glucose and serum creatinine, decrease urinary albumin, alleviate renal and colonic inflammation, and attenuate diabetic nephropathy lesions
Diabetic kidney disease (DKD) rats	Intragastric intervention with Moutan Cortex polysaccharides (MC-Pa); low-dose and high-dose groups were established with AG as control; continuous intervention for 12 weeks	Zhang et al. (2022d)	MC-Pa: 80 mg/kg BW (low dose), 160 mg/kg BW (high dose)	Animal experiment	Verrucomicrobia, Mollicutes, Bacteroidia, Muribaculaceae	—	Alleviate hyperglycemia and renal injury, reconstruct gut microbiota structure, improve intestinal barrier function, reduce serum pro-inflammatory mediators, elevate SCFA contents, and exert potential prebiotic effects
Cisplatin-treated mice	After cisplatin exposure, mice received intragastric administration of alginate oligosaccharides (AO) for 3 consecutive weeks; normal control (NC) group and cisplatin model (CIS) group without AO intervention were set as controls	Zhang et al. (2022c)	Cisplatin: 10 mg/kg *via* intraperitoneal injection; Alginate oligosaccharides: 5 mg/mL, 0.2 mL per single gavage	Animal experiment	*Lactobacillus johnsonii, Bacteroides vulgatus*	*Faecalibacterium, Bifidobacterium suis, Lactobacillus reuteri ilealis*	Ameliorate hepatic inflammation and renal oxidative damage induced by cisplatin chemotherapy, and regulate the composition of gut microbiota

### 5.4 Regulation of gut microbiota in kidney diseases by traditional Chinese medicine compound prescriptions

Owing to the unique advantages of multi-component, multi-target and multi-pathway properties, traditional Chinese medicine (TCM) compound prescriptions correct gut microbiota structural imbalance and exert multi-dimensional renoprotective effects *via* the gut-kidney axis. In recent years, numerous TCM compounds have shown defined gut microbiota-modulating activities across various kidney disease subtypes and preclinical animal models. Although their pharmacological mechanisms have respective emphases, they generally follow core principles: enrichment of beneficial bacteria, suppression of pathogenic bacteria, restoration of intestinal barrier function, and blockade of inflammatory and fibrotic signaling pathways.

In the clinical intervention of early-stage chronic kidney disease (CKD), 4-month combined treatment with Yishen Huashi Granules (YSHS) and RAAS inhibitors elevates abundances of short-chain fatty acid (SCFA)-producing bacteria such as *Faecalibacterium* and Lachnospiraceae, reduces levels of pro-inflammatory bacteria including *Eggerthella*, and concurrently decreases 24-h urinary protein excretion. KEGG functional enrichment analysis indicates that its therapeutic effects may be closely associated with regulation of N-glycan biosynthesis, lipid metabolism and vitamin metabolism pathways (Dong et al., 2024). For idiopathic membranous nephropathy (IMN), randomized controlled trials have verified that 6-month intervention with Jianpi Qushi Formula (JPQSF) induces characteristic alterations in gut microbial alpha and beta diversity, bringing overall microbial composition closer to that of healthy individuals, accompanied by reduced proteinuria and elevated plasma albumin levels (Lang et al., 2020). In CKD rat models, Jianpi Yishen Decoction (JPYS) specifically enriches butyrate-producing genera *Coprococcus* and *Akkermansia muciniphila*. Its urinary albumin-lowering efficacy differs from that of renal-targeted drugs, suggesting that intestinal butyrate-producing microbiota acts as a crucial mediator of its renoprotective actions (Zheng et al., 2020).

In terms of end-stage renal disease-related complications, Suyin Jiedu Granules downregulate pro-inflammatory bacteria and trimethylamine N-oxide (TMAO)-synthesis-related microbiota such as Muribaculaceae and *Bacteroides*, and upregulate *Lactobacillus* abundance in CKD rats. These changes reduce circulating TMAO levels, inhibit ferroptosis and profibrotic factor secretion in renal tubular epithelial cells, and further slow renal tubulointerstitial fibrosis progression (Ge et al., 2024). Although Tangshen Formula (TSF) does not alter the Firmicutes/Bacteroidetes (F/B) ratio in diabetic kidney disease (DKD) rats, it reverses the increased Bacteroidetes/Actinobacteria ratio, elevates *Bifidobacterium* abundance, and reduces levels of indoxyl sulfate (IS)-associated *Escherichia*. It thereby suppresses the aryl hydrocarbon receptor (AHR) pathway and the downstream JNK/NF-κB cascade of TLR4 to achieve renoprotection (Zhao et al., 2020). Qing-Re-Xiao-Zheng Formula (QRXZF) reverses the abnormally elevated F/B ratio induced by high-fat diet in early DKD mice, reduces LPS-producing microbiota such as Desulfovibrionaceae, and enriches SCFA-producing and beneficial microbes including *Aristipes* and *Akkermansia*. It further blocks activation of the LPS/TLR4/NF-κB inflammatory pathway by lowering serum LPS concentrations (Gao et al., 2021).

In clinical research, multicenter randomized trials focusing on DKD patients show that Zicui Yin (ZCY) has superior efficacy to Huangkui Capsules in reducing serum creatinine and improving estimated glomerular filtration rate (eGFR). Meanwhile, it increases abundances of SCFA-producing bacteria from Prevotellaceae and Lactobacillaceae, reduces levels of uremic toxin-generating microbiota such as Enterobacteriales and Clostridiaceae, and downregulates the F/B ratio (Liu J. et al., 2022). Additionally, combined administration of *Astragalus membranaceus* and *Salvia miltiorrhiza* reverses the elevated F/B ratio, enriches butyrate-producing *Akkermansia* and lactic acid-producing *Lactobacillus*, regulates butyric acid and tryptophan metabolic pathways, and alleviates renal metabolic disturbance and fibrotic progression in cyclosporine A-induced nephrotoxic rats (Han et al., 2021) (Table 2).

**TABLE 2:** Effects of traditional Chinese medicine compound prescriptions on gut microbiota in kidney diseases.

Patient/animal model	Intervention protocol	References	Dosage	Study type	Changes in relative abundance of gut microbiota		Main effects
					Increased	Decreased	
120 patients with chronic kidney disease (CKD)	Intervention group received RAAS inhibitors combined with Yishen Huashi (YSHS) Granules; control group received RAAS inhibitors alone; intervention lasted for 4 months	Dong et al. (2024)	One sachet of YSHS Granules, three times daily	Single-center randomized controlled trial	*Faecalibacterium*, Lachnospiraceae, *Lachnoclostridium, Sutterella, Bacteroides, Megamonas*	*Eggerthella, Clostridium innocuum*	Significantly reduce 24-h proteinuria levels in CKD patients, correct gut microbiota dysbiosis, and regulate glycan, lipid and vitamin metabolic pathways. Changes in gut microbial abundance are significantly correlated with the improvement of proteinuria
30 patients with idiopathic membranous nephropathy (IMN)	IMN patients received 6-month treatment with Jianpi Qushi Formula (JPQSF, TCM group) or immunosuppressants (western medicine group)	Lang et al. (2020)	Oral administration of JPQSF twice daily	Randomized controlled clinical trial	Characteristic abundance alterations of *Butyricimonas, Bacteroides, Alistipes* and Lachnospiraceae were observed after JPQSF intervention		JPQSF regulates gut microbiota composition and ameliorates microbial dysbiosis in IMN patients
CKD model rats	Intervention with Jianpi Yishen Formula (JPYS) and renal-targeted drug piperazine ferulate (PF); differences in gut microbial responses to the two treatments were compared; continuous intervention for 12 weeks	Zheng et al. (2020)	Daily intragastric dose: JPYS 10.89 mg/kg; PF 50 mg/kg	Animal experiment	Enriched *Coprococcus, Phascolarctobacterium, Parasutterella, Clostridium_XIVb*	Proteobacteria	JPYS markedly reduces urinary albumin, restores reticulocyte count and serum calcium level, and improves CKD-associated anemia and mineral bone metabolism disorders
CKD model rats	*In vitro* intervention of TMAO- and RSL3-induced NRK-52E cell injury with freeze-dried powder of Suyin Jiedu Formula; *in vivo* model established *via* adenine combined with high-choline diet, followed by 8-week intervention with Suyin Jiedu Granules (SDG) and TMAO inhibitor DMB	Ge et al. (2024)	Daily intragastric dose: SDG low dose 3.3 g/kg/d, high dose 6.6 g/kg/d	Animal experiment	Reverse the Firmicutes/Bacteroidetes community shift, downregulate TMAO-related genera and restore *Lactobacillus* abundance		SDG reduces circulating TMAO levels by remodeling gut microbiota structure and downregulating the abundance of TMAO-associated microbial taxa
Diabetic nephropathy rats	Continuous intervention with Tangshen Formula (TSF) or vehicle control for 12 weeks	Zhao et al. (2020)	Daily intragastric dose: TSF preparation 1.36 g/kg/d (crude drug equivalent 4.25 g/kg/d)	Animal experiment	Actinobacteria, Bifidobacteriaceae	Indoxyl sulfate precursor-producing *Escherichia, Anaerovibrio*, Enterobacteriaceae within Proteobacteria, and Ruminococcaceae within Firmicutes	TSF markedly alleviates renal pathological damage, reduces urinary albumin excretion, remodels gut microbiota dysbiosis, decreases the production of indoxyl sulfate and LPS, and downregulates renal and serum levels of inflammatory mediators including MCP-1 and TNF-α
Diabetic nephropathy rats	Control group fed with standard diet; DKD group and QRXZF group received high-fat diet for 7 weeks, followed by intraperitoneal injection of STZ for 5 consecutive days to establish the model	Gao et al. (2021)	Daily intragastric dose: QRXZF 15.6 g/kg/d	Animal experiment	Bacteroidetes, *Parasutterella*	Firmicutes, Proteobacteria, Verrucomicrobia, Desulfovibrionaceae, *Desulfovibrio*	QRXZF reduces urinary albumin, serum cholesterol and triglyceride levels in DKD mice, alleviates renal pathological injury, improves intestinal barrier integrity, reverses gut microbiota dysbiosis and decreases gut-derived LPS release
88 patients with diabetic kidney disease	Patients treated with Huangkui Capsules or Zicui Yin (ZCY) for 8 weeks; clinical and microbial indicators collected every 2 weeks during follow-up	Liu et al. (2022b)	ZCY: 75 g crude herb decocted to 150 mL, orally administered twice daily; Huangkui Capsules: 2.5 g per dose, three times daily (0.5 g per capsule)	Multicenter, parallel-controlled, open-label randomized clinical trial	Prevotellaceae, Lactobacillaceae	Enterobacteriales, Clostridiaceae, Micrococcaceae	ZCY effectively protects and improves renal function in DKD patients, delays eGFR decline and reduces serum creatinine levels
Cyclosporine A-induced chronic nephrotoxicity (CICN) model	Normal group received olive oil gavage; model group established with cyclosporine A; AS group administered cyclosporine A plus Astragalus-Salvia compound formula; FMT-AS group received fecal supernatant from AS group during modeling; drug intervention for the first 6 weeks and fecal microbiota transplantation for the subsequent 6 weeks	Han et al. (2021)	Daily intragastric dose: Astragalus-Salvia (AS) compound 8.4 g/kg/d (crude drug concentration 0.84 g/mL)	Animal experiment	*Akkermansia, Lactobacillus,* Lachnospiraceae, *Faecalibacterium, Roseburia*	Harmful genus *Holdemania*	Downregulate the level of metabolites that aggravate renal damage, ameliorate glomerular basement membrane thickening, renal tubular vacuolar degeneration and collagen hyperplasia, and reduce intestinal permeability
42 patients with peritoneal dialysis-related peritonitis (PDRP)	Patients assigned to blank control group, oral probiotic group and oral Fushen Granules group; intervention course lasted 8 weeks	Lin et al. (2021b)	Fushen Granules: 18 g per dose, twice daily; Bifidobacterium probiotics: 2 g per dose, three times daily after meals	Randomized controlled clinical trial	Bacteroidetes, *Bacteroides, Megamonas, Roseia,* Coxiellaceae*_bacterium_RFE02, Harryflintia*	Firmicutes	Fushen Granules elevate hemoglobin levels and reduce blood urea nitrogen and serum creatinine; correct gut microbiota dysbiosis by enriching beneficial bacteria related to carbohydrate and amino acid metabolism
29 peritoneal dialysis (PD) patients complicated with constipation	PD patients received oral intervention with Tiaopi Xiezhuo Formula (TXD) for 3 months	Peng et al. (2023)	TXD: 3 g crude herb equivalent per dose, orally administered twice daily	Clinical observational study	Enriched *Prevotella, Fusobacterium necrophorum*, specific strains of Lachnospiraceae, *Veillonella parvula, Paraprevotella clara*		TXD exerts no obvious effect on serum biochemical indicators of PD patients, but significantly relieves constipation, reduces abdominal distension incidence, increases soft stool proportion and completely eliminates hard stool symptoms

### 5.5 Gut microbiota modulation and renoprotective effects of plant polyphenols

Plant polyphenols are a large class of secondary metabolites widely distributed in natural plants, with well-documented antioxidant, anti-inflammatory and intestinal microecology-regulating activities. In recent years, multiple polyphenolic compounds including luteolin, resveratrol, epigallocatechin gallate (EGCG), curcumin and punicalagin have been confirmed to exert synergistic renoprotective effects in various chronic kidney disease (CKD) models *via* targeted regulation of the gut microbiota-gut-kidney axis.

#### 5.5.1 Luteolin (LUT)

Luteolin (LUT) is abundant in vegetables and medicinal plants, and its renoprotective efficacy has been validated in animal models of diabetic kidney disease, metabolic syndrome-related renal injury and lupus nephritis (Zhu et al., 2024; Guo et al., 2022; Ge et al., 2020). LUT repairs impaired intestinal barriers by upregulating expression of intestinal tight junction proteins including Claudin-1 and ZO-1(178), and simultaneously remodels gut microbiota composition. It elevates abundances of beneficial bacteria such as Bifidobacteriaceae and Ruminococcaceae, enriches short-chain fatty acid (SCFA)-producing microbes including *Ruminococcus*, and inhibits proliferation of lipopolysaccharide (LPS)-generating bacteria such as *Oscillospira* (Deshmukh et al., 2025; Pan et al., 2024). Apart from luteolin itself, its derivatives also have intestinal microecology-modulating and intestinal protective properties, which lay a foundation for their indirect renoprotective actions. Isoorientin (ISO), a 6-C-glucoside derivative of luteolin, suppresses growth of inflammation-associated pathogenic bacteria including *Acinetobacter baumannii*, *Desulfovibrio*, *Alternaria* and *Helicobacter*, while enriching beneficial genera such as *Bacteroides* and *Roseburia* to restructure the gut microbial community. Microbial metabolic pathway alterations mediated by isoorientin are positively correlated with host antioxidant, anti-inflammatory and antibacterial capacities, which further indirectly support renal protection by maintaining normal intestinal function (Yua et al., 2018).

#### 5.5.2 Resveratrol (RES)

Resveratrol (RES) is a natural polyphenol enriched in grapes, mulberries, red wine and peanut skins. With multiple pharmacological properties including potent antioxidant, anti-inflammatory, anti-fibrotic and gut microbiota-regulating activities, resveratrol exerts multi-target renoprotective effects in diabetic kidney disease, renal fibrosis and hypertension-associated renal damage (Meng et al., 2021). RES remodels gut microbiota structure to enrich SCFA-producing beneficial bacteria such as *Alistipes*, *Blautia*, *Lactobacillus* and *Faecalibaculum*, and reduce the abundance of opportunistic pathogens including *Eisenbergiella* and Desulfovibrionaceae, thereby regulating the SCFA-GPR receptor signaling axis or inhibiting the aryl hydrocarbon receptor pathway (Hsu et al., 2021; Tain et al., 2024; Zhou et al., 2024). Fecal microbiota transplantation experiments further verify that the anti-renal fibrosis effect of resveratrol is dependent on the gut microbiota-SCFA axis. Meanwhile, resveratrol repairs intestinal mucosal barriers, reduces LPS leakage, inhibits TLR4/NF-κB pathway activation, and alleviates local renal and systemic inflammatory responses. Its ester derivatives such as resveratrol butyrate also exert dual hypotensive and renoprotective effects by regulating gut microbiota and improving nitric oxide bioavailability (Yan et al., 2024; Cai et al., 2020).

#### 5.5.3 Epigallocatechin gallate (EGCG)

As the most abundant polyphenol in green tea, epigallocatechin gallate (EGCG) exerts established renoprotective effects in hyperuricemic nephropathy, diabetic kidney disease and lupus nephritis (Zhao T. et al., 2022; Kanla et al., 2019; Cai et al., 2018; Farhan, 2022). In hyperuricemia (HUA) mouse models, EGCG ameliorates uric acid metabolism *via* modulating gut microbiota composition, upregulates the expression of renal uric acid secretory transporter genes *Oat1* and *Oct1*, and downregulates the expression of uric acid reabsorption transporter genes *Urat1* and *Glut9*, thus alleviating renal injury from the perspective of uric acid transport regulation. EGCG reshapes gut microbial structure: it increases the abundances of Actinobacteriota and Bacteroidota at the phylum level; at the genus level, it enriches beneficial microbes including *Bifidobacterium*, Coriobacteriaceae UCG-002, norank_f_Muribaculaceae and *Faecalibaculum*, while decreasing *Lactobacillus* abundance. Correlation analyses further demonstrate that the abundances of Coriobacteriaceae, *Bifidobacterium* and norank_f_Muribaculaceae are positively correlated with *Oat1* expression, and *Oct1* expression is negatively associated with *Lactobacillus* abundance, which clarifies the specific mechanism by which EGCG regulates uric acid transport through gut microbiota modulation (Yu et al., 2024b).

The renoprotective activity of EGCG is highly dependent on its intact chemical structure, which has been confirmed in ICR mouse models. In this model, EGCG reduces plasma and urinary levels of the uremic toxin p-cresol (PC) in a concentration-dependent manner. Nevertheless, such protective effects are completely abolished after EGCG is hydrolyzed into epigallocatechin (EGC) and gallic acid (GA), indicating that the intact chemical structure is an essential prerequisite for its pharmacological functions (Unno and Ichitani, 2022). In terms of microbiota regulation, EGCG lowers the relative abundance of Firmicutes and Clostridiales, the core microbial taxa responsible for tyrosine metabolism and endogenous PC synthesis, thereby directly reducing microbial PC production. In addition, it elevates the abundances of Bacteroidetes and Verrucomicrobia, and increases the proportion of Bacteroidales at the order level, which optimizes intestinal microecology and reduces gut-derived toxin generation (Unno and Ichitani, 2022).

#### 5.5.4 Curcumin (CUR)

Curcumin (CUR) is a natural polyphenolic compound extracted from *Curcuma longa*, and its main active forms include curcumin, bisdemethoxycurcumin, demethoxycurcumin and tetrahydrocurcumin. Its renoprotective mechanisms have been well verified in animal models of hyperuricemic nephropathy, renal fibrosis and diabetic kidney disease (Huang et al., 2026). In rats with hyperuricemic nephropathy induced by combined administration of adenine and potassium oxalate, curcumin precisely modulates gut microbiota composition, inhibits excessive proliferation of opportunistic pathogens such as *Escherichia-Shigella* and *Bacteroides*, and elevates the relative abundance of SCFA-producing bacteria including *Lactobacillus* and Ruminococcaceae, thus alleviating renal injury indirectly by restoring intestinal microecological homeostasis (Xu X. et al., 2021). Curcumin also exerts anti-fibrotic and anti-inflammatory effects in 5/6 nephrectomy-induced renal fibrotic rats. It positively remodels gut microbiota and enriches beneficial genera including Lachnospiraceae_NK4A136_group, Eubacterium_siraeum_group and Muribaculaceae. Spearman correlation analyses reveal that these curcumin-enriched microbial genera are positively correlated with vitamin D and SCFA levels, and negatively correlated with biomarkers of chronic renal damage. At the molecular level, curcumin suppresses the activation of the LPS/TLR4/NF-κB and TGF-β1/Smads signaling pathways to synergistically inhibit renal fibrosis progression (Li et al., 2025b). As an important subtype of CKD, diabetic nephropathy (DN) can also be intervened by curcumin derivatives such as Zn(II)-curcumin, which exerts renoprotective effects *via* gut microbiota regulation. Zn(II)-curcumin ameliorates renal dysfunction, renal pathological lesions, inflammatory responses and disrupted zinc homeostasis. Fecal microbiota transplantation assays confirm that its regulatory effects on renal function, inflammation status and zinc homeostasis are partially microbiota-dependent, providing direct functional evidence that curcumin derivatives alleviate heavy metal-aggravated diabetic nephropathy through microbiota-mediated pathways (Sun et al., 2024).

Accumulating clinical evidence further supports the gut microbiota-regulating and gut-kidney axis-protective properties of curcumin. Six-month oral supplementation of curcumin (Meriva formulation) optimizes fecal microbial composition in CKD patients, manifested as decreased abundance of *Escherichia-Shigella* and increased level of *Lachnoclostridium*. Moreover, the abundance of Lactobacillaceae spp. rises in the later 3 months of intervention, suggesting that curcumin achieves long-term renoprotection *via* sustained regulation of gut microbial homeostasis (Pivari et al., 2022). Beyond gut microbiota and intestinal barrier modulation, recent studies have identified a novel mechanism that curcumin upregulates intestinal alkaline phosphatase (IALP) expression to further strengthen gut-kidney axis protection. As a key ectoenzyme on the intestinal luminal surface, IALP exerts detoxification effects on LPS *via* dephosphorylation, relieves LPS-induced intestinal mucosal barrier damage and inhibits the initiation of gut-derived systemic inflammation, which fundamentally blocks the translocation of intestinal toxins into the kidney and provides a solid theoretical basis for the clinical application of curcumin in CKD management (Santos et al., 2022; Ghosh et al., 2014).

#### 5.5.5 Punicalagin (PU)

Punicalagin (PU) is the predominant ellagitannin polyphenolic active ingredient extracted from pomegranate and pomegranate peel. It has strong antioxidant activity, gut microbiota-modulating capacity and broad-spectrum anti-inflammatory properties, and shows promising application prospects for intervention in chronic renal injuries including diabetic kidney disease (Hua et al., 2022; Xu J. et al., 2021; Han et al., 2025).

In HUA mouse models, punicalagin intervention triggers characteristic gut microbiota remodeling and reverses HUA-induced microbial dysbiosis. At the phylum level, it increases Bacteroidota abundance and reduces Firmicutes abundance as well as the Firmicutes/Bacteroidetes (F/B) ratio. At the genus level, it restrains proliferation of pro-inflammatory bacteria such as *Parabacteroides*, *Oscillibacter* and *Desulfovibrio*. These pro-inflammatory microbes aggravate intestinal mucosal barrier damage and renal inflammatory responses, and their suppression by punicalagin reduces the inflammatory burden in both intestine and kidney at the source. Meanwhile, PU elevates abundances of SCFA-producing bacteria including Prevotellaceae_UCG-001 and norank_f_Muribaculaceae, promotes intestinal SCFA synthesis and inhibits LPS production (Han et al., 2025). In addition, punicalagin reverses gut microbial disorders in high-fat diet-induced diabetic mice: it reduces the intestinal F/B ratio and Proteobacteria abundance to mitigate intestinal microecological destruction caused by harmful bacteria, and increases levels of SCFA-producing bacteria such as *Akkermansia* and Eubacterium_coprostanoligenes_group. High-dose punicalagin specifically enriches Lachnospiraceae, another category of SCFA-producing microbes, to further optimize the metabolic function of gut microbiota (Hua et al., 2022) (Table 3).

**TABLE 3:** Effects of plant polyphenols on gut microbiota and renal protection.

Plant polyphenol	Patient/animal model	Intervention protocol	References	Dosage	Study type	Changes in relative abundance of gut microbiota		Main effects
						Increased	Decreased	
Luteolin (LUT)	Diabetic nephropathy rats	Pioglitazone group and low-/high-dose HGGG groups received daily intragastric administration for 8 consecutive weeks	Pan et al. (2024)	Pioglitazone 20 mg/kg/d; HGGG low dose 200 mg/kg/d, high dose 400 mg/kg/d, daily gavage	Animal experiment	Increased abundance of Actinobacteriota; corrected the imbalance of Firmicutes and Bacteroidetes; elevated abundances of Bifidobacteriaceae and Ruminococcaceae; enriched SCFA- and polyamine metabolism-related beneficial bacteria including Anaerovoracaceae, Prevotellaceae NK3B31 and *Barnesiella_sp*	LPS-producing pathogenic bacteria such as *Oscillospira* and harmful *Clostridium*	Reduce UACR, blood urea nitrogen and urinary NAG levels in diabetic nephropathy rats; alleviate renal fibrosis and inhibit NLRP3 inflammasome activation; improve intestinal barrier integrity and remodel gut microbiota structure
	Metabolic syndrome rat model	Control group fed with low-fat diet; other groups fed with high-fat diet providing 60% of total energy; simultaneous daily intragastric intervention with peanut shell extract (PSE) and luteolin for 4 months	Deshmukh et al. (2025)	PSE 200 mg/kg/d, LUT 100 mg/kg/d, oral gavage	Animal experiment	Bacteroidetes, *Phocaeicola vulgatus, Marinibacterium*	-	Ameliorate high-fat diet-induced glucose metabolism disorder, insulin resistance and pancreatic β-cell function impairment; reduce serum lipopolysaccharide-binding protein levels; partially reverse gut microbiota dysbiosis induced by high-fat diet; relieve chronic inflammation, oxidative stress and mitochondrial dysfunction associated with metabolic syndrome
Resveratrol (RES)	CKD rat model	Normal group fed with standard diet; control group treated with resveratrol for 6 weeks	Tain et al. (2024)	Resveratrol 50 mg/L	Animal experiment	Increased abundances of *Clostridium, Bacteroides, Eubacterium, Ruminococcus, Ligilactobacillus* and *Ligilactobacillus murinus*	-	Improve *in vivo* nitric oxide bioavailability and upregulate plasma SCFA levels; alleviate hypertension induced by chronic kidney disease *via* remodeling gut microbiota and ameliorating NO and SCFA metabolic disorders
	CKD rat model	Normal group fed with standard diet; control group treated with resveratrol for 6 weeks	Hsu et al. (2021)	Resveratrol 50 mg/L (10 mg/kg/d)	Animal experiment	Actinobacteriota, *Parabacteroides, Alistipes, Escherichia, Akkermansia, Blautia, Enterococcus*	-	Reduce serum creatinine and blood pressure in CKD rats; ameliorate renal hypertrophy and creatinine clearance rate; protect rats against hypertension and renal injury
	Hyperuricemic rat model	Low-fat diet control group, high-fat diet (HFD)-induced hyperuricemia (HUA) model group, and HFD plus resveratrol (RES) intervention group; intervention lasted 8 weeks for each group	Zhou et al. (2024)	RES supplemented at 0.1% in diet, equivalent to approximately 100 mg/kg/day in mice	Animal experiment	Lactobacillaceae	Prevotellaceae, Desulfovibrionaceae	Reduce serum uric acid, creatinine, blood urea nitrogen and urinary protein levels in HUA mice; ameliorate glomerular atrophy, abnormal renal tubular structure, renal fibrosis and renal inflammation
	Diabetic nephropathy mice	All groups fed with standard diet; db/db mice received regular diet or resveratrol-containing diet for 12 weeks	Yan et al. (2024)	Resveratrol supplemented at 0.4% in diet	Animal experiment	Firmicutes, Lactobacillaceae, *Lactobacillus, Faecalibacterium*	Lachnospiraceae, Oscillospiraceae and Lachnospiraceae NK4A136 group	Delay the progression of diabetic nephropathy and alleviate renal interstitial fibrosis; remodel gut microbiota dysbiosis in db/db mice; enrich SCFA-producing beneficial bacteria and elevate fecal acetate content
	Diabetic nephropathy mice	All mice fed with standard diet; resveratrol group received daily intragastric administration, and control group treated with equal volume of normal saline synchronously for 12 consecutive weeks	Cai et al. (2020)	Resveratrol 10 mg/kg/d, daily gavage	Animal experiment	Bacteroidetes, *Bacteroides, Alistipes, Parabacteroides*	Firmicutes	Repair intestinal barrier and alleviate intestinal inflammation; effectively reverse gut microbiota dysbiosis in db/db mice
	CKD rats with cognitive impairment	CKD cognitive impairment model established by two interval intravenous injections of doxorubicin; resveratrol administered by gavage for 6 weeks after modeling	Shao et al. (2025)	Doxorubicin 4 mg/kg; RES 50 mg/L, RBE 25 mg/L and 50 mg/L, RPE2 25 mg/L, RPE4 25 mg/L	Animal experiment	*Clostridium sensu stricto, Bifidobacterium, Romboutsia, Alistipes, Blautia, Parabacteroides, Akkermansia, Enterococcus, Eubacterium, Allobaculum, Ruminococcus, Ligilactobacillus*	*Butyricicoccus, Ruminococcus_1,Parabacteroides*	Ameliorate renal dysfunction, glomerular atrophy and pathological damage in colon and hippocampus of doxorubicin-induced CKD rats; relieve cognitive decline; reduce serum inflammatory factors and LPS levels; inhibit activation of hippocampal TLR4/NFκB inflammatory pathway; correct gut microbiota imbalance and suppress microbial LPS biosynthesis
Epigallocatechin gallate (EGCG)	Hyperuricemic mice	Intervention with epigallocatechin gallate (EGCG); allopurinol (AP) set as positive control	Yu et al. (2024b)	EGCG 50 mg/kg	Animal experiment	Actinobacteriota, Bacteroidota, *Bifidobacterium*, Coriobacteriaceae UCG-002, norank_f_Muribaculaceae, *Faecalibacterium*	*Lactobacillus*	Inhibit intestinal tyrosine metabolism and reduce synthesis and accumulation of uremic toxin p-cresol; alleviate hyperuricemia and exert renoprotective effects
	Chronic kidney disease mice	Diet supplemented with gradient concentrations of EGCG; meanwhile, diet containing EGCG enzymatic hydrolysates EGC + GA set as control; free access to food and water for 2 weeks	Unno and Ichitani (2022)	Diet supplemented with 0.05%, 0.1%, 0.2% (w/w) EGCG respectively	Animal experiment	Bacteroidetes, Verrucomicrobia, Bacteroidales	Firmicutes, Clostridiales	Intact EGCG structure inhibits intestinal microbial tyrosine metabolism and reduces microbial synthesis of p-cresol; remodel intestinal microecology and alleviate uremic toxin accumulation
Curcumin	Hyperuricemic mice	Modeling after adaptive feeding; curcumin administered by daily gavage for 8 weeks	Xu et al. (2021a)	Curcumin 200 mg/kg, daily gavage	Animal experiment	Increased abundances of *Lactobacillus, Alloprevotella, Ruminococcus_1* and Ruminococcaceae_UCG_014	Reduced abundances of *Escherichia-Shigella* and *Bacteroides*	Significantly reduce serum uric acid, creatinine, blood urea nitrogen and metabolic endotoxin levels; alleviate renal pathological damage
​	24 patients with stage 3a–4 chronic kidney disease (CKD)	CKD patients received nutritional counseling plus curcumin phospholipid complex intervention for 3 and 6 months	Pivari et al. (2022)	Curcumin phospholipid complex 500 mg per tablet, twice daily	Pilot clinical study	*Lachnoclostridium, Lachnospira,* Lactobacillaceae	*Escherichia-Shigella,* Enterobacteriaceae, Verrucomicrobia	Significantly reduce plasma pro-inflammatory mediators and lipid peroxidation levels; regulate gut microbiota composition; ameliorate inflammatory and oxidative status in CKD patients
	Chronic kidney disease rats	Curcumin suspension administered by daily gavage; sham operation group and model group treated with equal volume of 0.1% CMC-Na vehicle for 12 consecutive weeks	Li et al. (2025b)	Curcumin 200 mg/kg, once daily gavage	Animal experiment	Bacteroidetes, Muribaculaceae, Lachnospiraceae_NK4A136_group, *Eubacterium_siraeum_group*	Erysipelotrichales, Sphingomonadales, NK4A214_group, Family_XIII_AD3011_group	Remodel intestinal microecology and enhance microbial diversity; improve intestinal tight junction protein expression and alleviate colonic inflammation; inhibit LPS/TLR4/NF-κB and TGF-β1/Smads pathways to relieve renal fibrosis and systemic inflammation
	Diabetic nephropathy rats	Model established *via* cadmium chloride exposure in drinking water combined with intraperitoneal STZ injection; rats treated with intragastric Zn(II)-curcumin, PVP and positive drug metformin respectively; blank control set and gut microbiota-mediated effects verified by fecal microbiota transplantation; continuous intervention for 8 weeks	Sun et al. (2024)	Zn(II)-curcumin 100 mg/kg/d	Animal experiment	Bacteroidetes, S24-7_group, *Prevotella_9*	Firmicutes, uncultured Ruminococcaceae	Remodel gut microbiota structure and serum metabolic profile in cadmium-exposed diabetic nephropathy rats; correct zinc homeostasis imbalance; suppress TLR4/NF-κB pathway activation; alleviate renal pathological damage and inflammatory response
Punicalagin (PU)	Hyperuricemic mice	Punicalagin (PU) administered by daily gavage; normal group and model group treated with 0.5% CMC-Na vehicle for 2 consecutive weeks	Han et al. (2025)	Punicalagin 100, 200, 300 mg/kg/d	Animal experiment	Prevotellaceae_UCG-001,Muribaculaceae	*Parabacteroides, Oscillibacter,Desulfovibrio, Tuzzerella*	Significantly reduce serum uric acid levels; relieve renal and intestinal injury induced by hyperuricemia
	Diabetic nephropathy mice	Fed with high-fat diet; long-term intervention with different doses of punicalagin (PU) for 8 consecutive weeks	Hua et al. (2022)	Punicalagin 50 mg/kg/d, 100 mg/kg/d	Animal experiment	*Akkermansia, Eubacterium_coprostanoligenes_group,* Lachnospiraceae	Elevated Firmicutes/Bacteroidetes ratio and Proteobacteria abundance	Remodel intestinal microecology and repair intestinal barrier; elevate cecal short-chain fatty acid levels; alleviate diabetic renal injury

Although luteolin, resveratrol, EGCG, curcumin, punicalagin and other plant polyphenols differ in molecular structures and primary functional orientations, they all exert renoprotective effects *via* a unified gut-kidney axis regulatory strategy covering microbiota remodeling, intestinal barrier restoration, inflammatory signal blockade and metabolic modulation. These findings fully demonstrate that plant polyphenols have substantial potential as promising therapeutic agents for kidney disease.

### 5.6 Interventional effects of other natural bioactive components

In addition to the aforementioned natural polysaccharides, traditional Chinese medicine compound prescriptions and plant polyphenols, a variety of other natural bioactive substances have been confirmed to exert renoprotective effects by regulating the gut microbiota-gut-kidney axis in recent years. These ingredients include coumarins, flavonoid glycosides, anthraquinones, terpenoids and other chemical classes, and they differ in their core mechanisms of action.

In studies of targeted aryl hydrocarbon receptor (AhR) pathway modulation, work by Li et al. has shown that justicinoside A (BSA) ameliorates renal function in chronic kidney disease (CKD) rats. This compound alleviates renal inflammation and podocyte damage by directly inhibiting AhR signaling activation and regulating the NF-κB and Nrf2 pathways. Likewise, increased abundance of *Lactobacillus johnsonii* elevates serum indole-3-aldehyde (IAId) levels to suppress AhR signaling in a microbiota-dependent manner and alleviate renal injury in CKD conditions. Both substances represent potential intervention strategies targeting the AhR pathway (Miao et al., 2024b; Miao et al., 2022b; Miao et al., 2020; Li et al., 2024).

In terms of gut microbial metabolite regulation, isoquercitrin (ISO) has been shown to inhibit intestinal bacterial complex I activity and reduce proton potential, thus interfering with tryptophan transmembrane transport. It further reduces indole biosynthesis and subsequent production of indoxyl sulfate (IS), a major uremic toxin, and ultimately relieves renal fibrosis (Wang et al., 2020). Madecassoside specifically promotes proliferation and increases intestinal abundance of *Bacteroides fragilis*. It reduces lipopolysaccharide (LPS) content and upregulates renal SGLT2 expression to enhance 1,5-AG reabsorption, thereby activating the TGR5 pathway, suppressing oxidative stress and inflammatory responses, improving renal function and attenuating renal tubulointerstitial fibrosis (Zhou et al., 2022). Neohesperidin increases intestinal abundance of *Bacteroides ovatus*. Via the regulatory effects of this species, it improves renal function, decreases serum creatinine and blood urea nitrogen levels, and mitigates renal tubulointerstitial fibrosis in mice with adenine-induced CKD and unilateral ureteral obstruction (UUO)-induced renal damage (Si et al., 2024). Moreover, coumarin mixtures (HP) isolated from *Hydrangea* correct gut microbiota dysbiosis and lower the abnormally elevated Firmicutes/Bacteroidetes (F/B) ratio in membranous nephropathy (MN) rats. These mixtures restore colonic epithelial barrier integrity, reduce synthesis of uremic toxin precursors including indole derivatives and p-cresyl glucuronide, and promote production of short-chain fatty acids (SCFAs) including acetate, propionate and butyrate, which helps optimize the intestinal microenvironment and relieve renal lesions in MN model animals (Li Z. et al., 2023).

For gut microbiota remodeling to suppress renal fibrosis, studies show that emodin-based enema preparations (EMO-IGE) exert anti-fibrotic effects in rats with UUO-induced renal fibrosis mainly *via* modulating gut microbial composition. These preparations reduce abundances of pathogenic bacteria such as *Escherichia-Shigella* and *Streptococcus*, and elevate levels of beneficial bacteria including *Bacteroides* and *Lactobacillus*, thus downregulating expression of fibrotic markers such as α-SMA and type I collagen (Xu et al., 2022). Astragalus aqueous extract (AAE) also reverses the abnormally increased F/B ratio and upregulates abundances of beneficial bacteria dominated by *Lactobacillus* in hyperuricemic nephropathy (HN) rats. It regulates the gut microbiota-SCFA metabolic axis, inhibits purine metabolism and inflammatory reactions, and alleviates renal inflammation and histopathological damage (Qu et al., 2025). Alisol B 23-acetate (ABA) reverses abnormal abundances of twelve hypertension-related intestinal bacterial genera in 5/6 nephrectomized rats to restore gut microbial balance and reduce blood pressure. Meanwhile, it selectively blocks profibrotic signaling pathways including TGF-β/Smad3 and Wnt/β-catenin to inhibit renal tubulointerstitial fibrosis progression (Chen et al., 2020).

## 6 Organoprotective effects of natural products in dialysis patients

### 6.1 Intervention efficacy in hemodialysis patients

For hemodialysis recipients, natural products mainly reduce uremic toxin burden and improve systemic inflammatory status by regulating gut microbial structure and toxin synthesis pathways. Clinical studies have confirmed that curcumin decreases plasma p-cresyl sulfate (PCS) levels in hemodialysis patients. This pharmacological effect is associated with increased intestinal *Lachnoclostridium* abundance and decreased *Escherichia-Shigella* abundance, achieving precise intervention at the source of toxin production (Pivari et al., 2022; Salaro et al., 2021).

Prebiotic-type natural ingredients also show promising intervention potential. High-amylose resistant starch HAM-RS2 lowers serum urea, interleukin-6 (IL-6), tumor necrosis factor-α (TNF-α) and malondialdehyde (MDA) levels in hemodialysis patients, relieves inflammation and oxidative stress, and alleviates constipation symptoms (Tayebi Khosroshahi et al., 2018). Compound prebiotics composed of fructooligosaccharide and inulin reduce gut-derived PCS production and decrease serum PCS concentrations *via* modulating gut microbial metabolism. Combined use of inulin and a low-protein diet elevates intestinal Bifidobacteriaceae abundance, reduces serum TNF-α, NOX2, uric acid and C-reactive protein levels in non-dialyzed CKD patients, and ameliorates microinflammation and metabolic disorders from multiple perspectives (Lai et al., 2019; Meijers et al., 2010). Nevertheless, not all natural products have definite therapeutic efficacy. Data from a randomized double-blind placebo-controlled trial indicate that 8-week intervention with Brazilian green propolis extract fails to reduce plasma indole-3-acetic acid, IS or PCS levels significantly, and does not induce statistically significant alterations in fecal microbial composition. These negative findings suggest that propolis may not reverse intestinal microecological disturbance in dialysis patients over a short intervention period. Nutritional strategies targeting the gut-kidney axis should be selected strictly based on high-quality clinical evidence, rather than assuming universal efficacy for all natural products with antibacterial or antioxidant properties (Fonseca et al., 2024).

### 6.2 Intervention efficacy in peritoneal dialysis patients

For peritoneal dialysis recipients, traditional Chinese medicine compound formulas show unique advantages in ameliorating peritonitis-related peritoneal fibrosis and relieving constipation. Research shows that Fushen Granules (FSG) delay peritoneal fibrosis by suppressing expression of inflammatory and fibrotic mediators including IL-6, TGF-β1, CTGF and VEGF in patients with peritoneal dialysis-related peritonitis (PDRP). In addition, FSG regulates gut microbiota to enrich *Bacteroides*, indirectly promote short-chain fatty acid synthesis and strengthen intestinal mucosal barrier function. It also reduces serum blood urea nitrogen and creatinine levels, raises serum albumin content, and improves nutritional status and renal function (Lin W. et al., 2021). Regarding constipation relief in peritoneal dialysis patients, a prospective clinical observation of 29 subjects has confirmed that 3 months of oral Tiaopi Xiezhuo Decoction (TXD) reduces the incidence of abdominal distension by 80%, increases the proportion of loose stools by 2.6-fold and completely eliminates hard stool symptoms. This formula restructures the intestinal microbial community, and enriched microbial species are strongly correlated with constipation improvement. TXD relieves constipation by correcting intestinal microecological imbalance and enriching specific beneficial bacteria (Peng et al., 2023).

## 7 Conclusion

This review systematically summarizes gut microbiota dysbiosis characteristics across various chronic kidney disease subtypes, including diabetic kidney disease, membranous nephropathy, lupus nephritis, IgA nephropathy, hypertension-complicated chronic kidney disease, and end-stage renal disease patients receiving hemodialysis or peritoneal dialysis. These diseases share consistent patterns of microecological disturbance, while presenting distinct microbial biomarker profiles corresponding to disease specificity, stage specificity and dialysis modality dependence.

Disruption of intestinal mucosal barrier integrity caused by gut microbial homeostasis breakdown facilitates entry of gut-derived uremic toxins—including lipopolysaccharide, indoxyl sulfate, p-cresyl sulfate and trimethylamine N-oxide—into the systemic circulation. These toxins further activate multiple signaling cascades including TLR4/NF-κB, Nrf2/ARE, AhR, NLRP3 inflammasome, ferroptosis and TGF-β/Smad pathways, triggering systemic microinflammation, oxidative stress dysregulation and renal tubulointerstitial fibrosis. This constitutes the core pathological loop through which the gut microbiota–gut–kidney axis participates in CKD onset, progression and dialysis-related complications, and provides a solid theoretical basis for elucidating novel pathogenic mechanisms and identifying targeted therapeutic targets for kidney disease.

Natural products represented by natural polysaccharides, traditional Chinese medicine compound formulas and plant polyphenols feature multi-component composition, multi-target activity, low toxicity and favorable biocompatibility. They exert systemic regulation of the gut–kidney axis and renoprotective effects through multiple pathways: restructuring gut microbial communities, enriching short-chain fatty acid-producing beneficial bacteria, inhibiting proliferation of pathogenic bacteria and uremic toxin-synthesizing microbes, restoring intestinal barrier function, and hierarchically regulating signaling pathways involved in inflammation, oxidative stress, cell death and fibrosis.

Natural polysaccharides mainly exert prebiotic-like effects, focusing on reshaping gut microbial structure and short-chain fatty acid metabolism. Traditional Chinese medicine compound formulas correct microecological disturbance and block inflammatory-fibrotic cascades *via* their holistic regulatory properties. Plant polyphenols have potent antioxidant, anti-inflammatory and bidirectional gut microbiota regulatory activities. Nevertheless, heterogeneity exists in the intervention effects of different natural products. Some compounds fail to deliver consistent clinical benefits, so the renoprotective effects of natural products cannot be generalized, and appropriate intervention agents should be precisely selected based on evidence-based medical data.

Current research in this field still has several limitations. Most relevant studies remain basic animal experiments, and only a small number of traditional Chinese medicine formulas and individual plant polyphenols have been evaluated in small-sample clinical trials. Large-scale, multicenter randomized controlled trials remain limited, resulting in an incomplete clinical evidence base. Most mechanistic studies only verify phenotypic changes in single signaling pathways or individual microbial taxa. The dose–response and structure–activity relationships of various natural products remain unclear. Few studies perform head-to-head efficacy comparisons or define optimal indications for different bioactive components. Individualized intervention strategies for different CKD subtypes, disease stages and specific dialysis populations remain lacking, which severely restricts clinical translation and large-scale application of gut–kidney axis-based prevention and treatment strategies using natural products.

As a narrative review, this work also has methodological limitations. Standardized systematic literature retrieval and screening procedures were not applied, quantitative meta-analysis was not performed, and assessment of literature quality and risk of bias for included studies is not provided. Despite these limitations, this review retains important academic reference value and clinical guiding significance.

Overall, targeted regulation of gut microecology and natural product-based intervention targeting the gut microbiota–gut–kidney axis hold substantial potential to break through existing therapeutic bottlenecks and innovate the full-course prevention and treatment system for chronic kidney disease, with broad application prospects.
